# T cells in cancer: mechanistic insights and therapeutic advances

**DOI:** 10.1186/s40364-025-00807-w

**Published:** 2025-07-15

**Authors:** Jingjing Pu, Ting Liu, Yi Zhou, Mengping Chen, Xuehang Fu, Yike Wan, Junying Wang, Binzhen Chen, Amit Sharma, Veronika Lukacs-Kornek, Ingo G.H. Schmidt-Wolf, Jian Hou

**Affiliations:** 1 Department of Hematology, Renji Hospital, Shanghai Jiao Tong University School of Medicine, Shanghai, Shanghai, 200127 China; 2 Department of Geriatrics, Renji Hospital, Shanghai Jiao Tong University School of Medicine, Shanghai, Shanghai, 200127 China; 3 Department of Integrated Oncology, Center for Integrated Oncology (CIO) Bonn, University Hospital Bonn, NRW, 53127 Bonn, Germany; 4 Institute of Molecular Medicine and Experimental Immunology, University Hospital Bonn, NRW, 53127 Bonn, Germany

**Keywords:** T cells, Cancer immunotherapy, Tumor microenvironment, Adoptive T cell therapy, Precision treatment

## Abstract

T cells are central players in the fight against cancer, capable of recognizing and destroying tumor cells. However, tumors often find ways to evade this immune response, creating challenges for effective treatment. In this review, we explore how different T cell subsets—including cytotoxic T cells, helper T cells, regulatory T cells, and unconventional T cells—contribute to tumor progression or suppression. We also delve into key mechanisms, such as immune checkpoints and metabolic pathways, that shape T cell behavior in the tumor microenvironment. Advances in cancer immunotherapy, including immune checkpoint inhibitors (ICIs), T cell engagers (TCEs), adoptive T cell therapies (ACTs), chimeric antigen receptor (CAR) T cell therapies, and cancer vaccines, have transformed cancer treatment and provided new hope for patients. However, challenges such as treatment resistance, limited efficacy in solid tumors, and therapy-associated toxicities remain significant barriers to broader clinical success. We discuss innovative strategies to tackle these challenges, including combination therapies and next-generation T cell engineering approaches. By connecting the biology of T cells with cutting-edge therapeutic advances, this review aims to inspire progress in the development of more effective and personalized cancer treatments.

## Introduction

The immune system plays a vital role in cancer control, with T cells serving as central mediators of anti-tumor immunity [1–3]. Through their ability to recognize and eliminate malignant cells via antigen-specific mechanisms, T cells form the foundation of cancer immunosurveillance [4–6]. However, the interaction between T cells and tumors is highly dynamic and complex [7–10]. Tumors employ various strategies to evade immune detection, including inducing T cell exhaustion [11–13], promoting regulatory T cell (Treg) activity [4, 14, 15], and reshaping the tumor microenvironment into an immunosuppressive niche [16–18]. These mechanisms not only impair T cell function but also enable tumor progression, posing significant challenges for effective cancer therapies.

Over the past decade, T cell-based therapies have transformed the oncology landscape [19–21]. Immune checkpoint inhibitors (ICIs), which reinvigorate exhausted T cells by blocking inhibitory pathways such as PD-1/PD-L1 and CTLA-4, have achieved remarkable success in treating certain cancers [22–26]. Similarly, chimeric antigen receptor (CAR) T cell therapy, which engineers patient T cells to specifically target tumor antigens, has shown unprecedented efficacy in hematologic malignancies [27–31]. These advancements highlight the therapeutic potential of leveraging T cells in cancer treatment. However, significant challenges remain. Many solid tumors demonstrate resistance to ICIs and CAR T cell therapy due to limited T cell infiltration, antigen heterogeneity, and an immunosuppressive tumor microenvironment (TME) [32–35]. Additionally, treatment-related toxicities, including cytokine release syndrome (CRS) and immune-related adverse events, further complicate the clinical application of these therapies [36–40]. Addressing these barriers requires a deeper understanding of the mechanisms regulating T cell behavior and the development of innovative therapeutic strategies.

This review provides an integrated perspective on the roles of T cells in cancer, focusing on their mechanistic contributions to tumor control and progression. It also highlights recent advancements in T cell-based immunotherapies, identifies key limitations of existing approaches, and explores emerging technologies aimed at improving therapeutic outcomes. By integrating mechanistic insights with clinical developments, this review aims to contribute to the advancement of more effective and personalized T cell-based therapies for cancer.

## Mechanistic insights into T cells in cancer

### Development of T cells

**Fig. 1:**
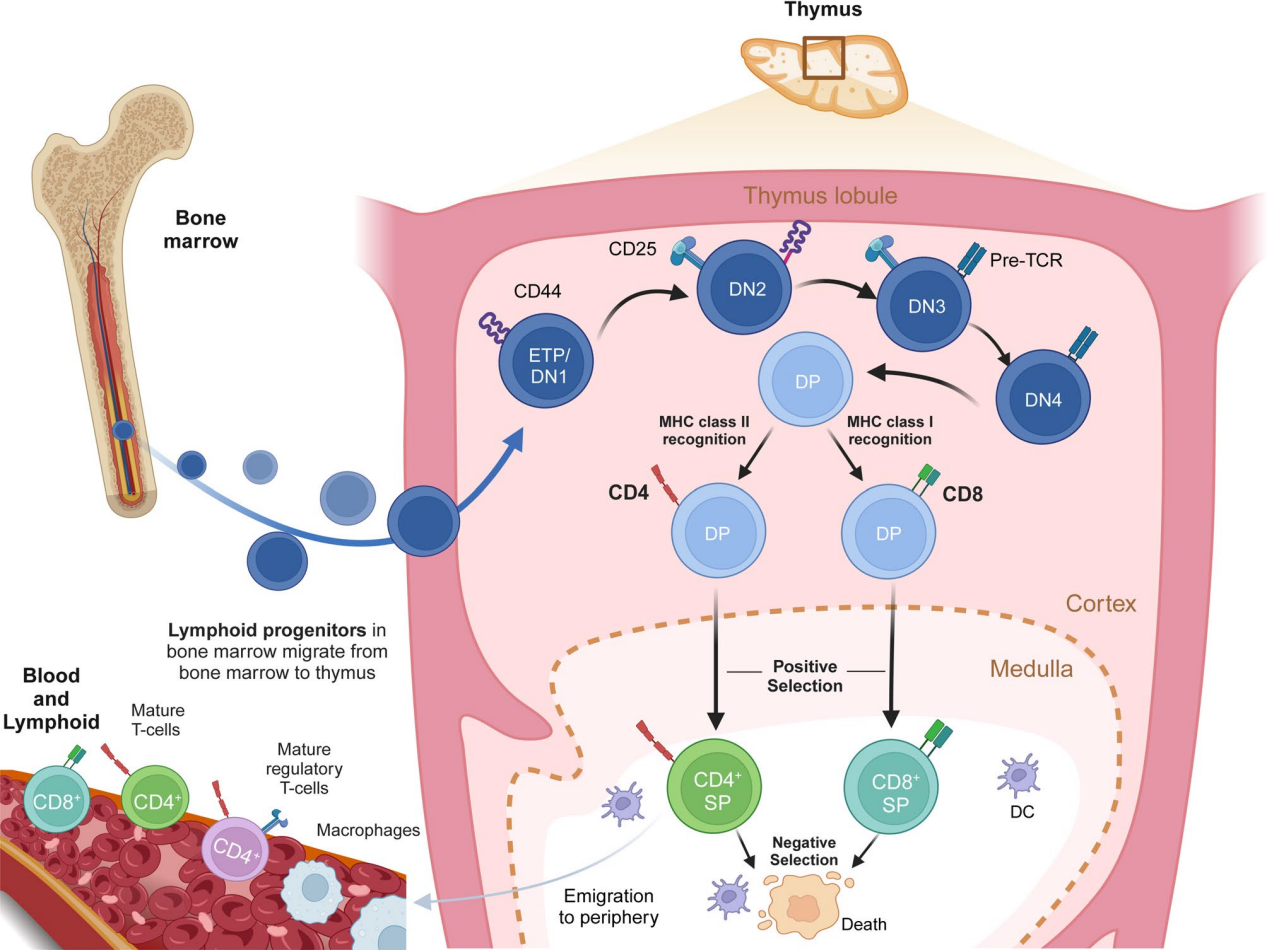
Overview of T-cell development in the thymus. Committed lymphoid progenitors originate in the bone marrow and migrate to the thymus, where they differentiate into DN thymocytes, lacking TCR, CD4, and CD8. DN thymocytes progress through four stages: early thymic progenitors (ETPs)/DN1 (CD44^+^CD25^−^), DN2 (CD44^+^CD25^+^), DN3 (CD44^−^CD25^+^), and DN4 (CD44^−^CD25^−^). During the DN2 to DN4 transition, thymocytes express the pre-TCR, consisting of a rearranged TCR β-chain and the invariant pre-Tα chain. Successful pre-TCR signaling drives proliferation and the replacement of the pre-TCR α-chain with a rearranged TCR α-chain, forming a complete αβ-TCR. DP thymocytes interact with MHC-expressing cortical epithelial cells presenting self-peptides. TCR signaling strength determines thymocyte fate: weak signaling causes death by neglect, strong signaling leads to negative selection, and optimal signaling induces positive selection. T cells that bind self-peptide–MHC class I become CD8^+^ T cells, while those binding MHC class II become CD4^+^ T cells, maturing for export to peripheral blood and lymphoid tissues. Figure created with BioRender.com

T cells, crucial for adaptive immunity, originate from hematopoietic stem cells in the bone marrow and migrate to the thymus for maturation [41–43] (Fig. 1). In the thymus, thymocytes undergo sequential stages, starting as double-negative (DN) cells lacking CD4 and CD8 markers, followed by T cell receptor (TCR) gene rearrangement [44–47]. These thymocytes then become double-positive (DP) cells, expressing both CD4 and CD8, where they undergo positive and negative selection. Positive selection ensures that T cells can recognize self-major histocompatibility complex (MHC) molecules, while negative selection eliminates T cells with high affinity for self-antigens, preventing autoimmunity [48–52]. After selection, thymocytes differentiate into single-positive (SP) cells, expressing either CD4 (helper T cells) or CD8 (cytotoxic T cells), and exit the thymus as mature T cells. These T cells circulate in peripheral blood and lymphoid tissues, acting as effector cells in immune responses or developing into memory T cells for long-term immunity [53, 54]. The maturation and selection of T cells are tightly regulated by cytokines such as interleukin-7 (IL-7) and transcription factors like T-bet and GATA3, ensuring the production of functional, self-tolerant T cells essential for immune defense [55–60].

### Differentiation of T cells

**Fig. 2:**
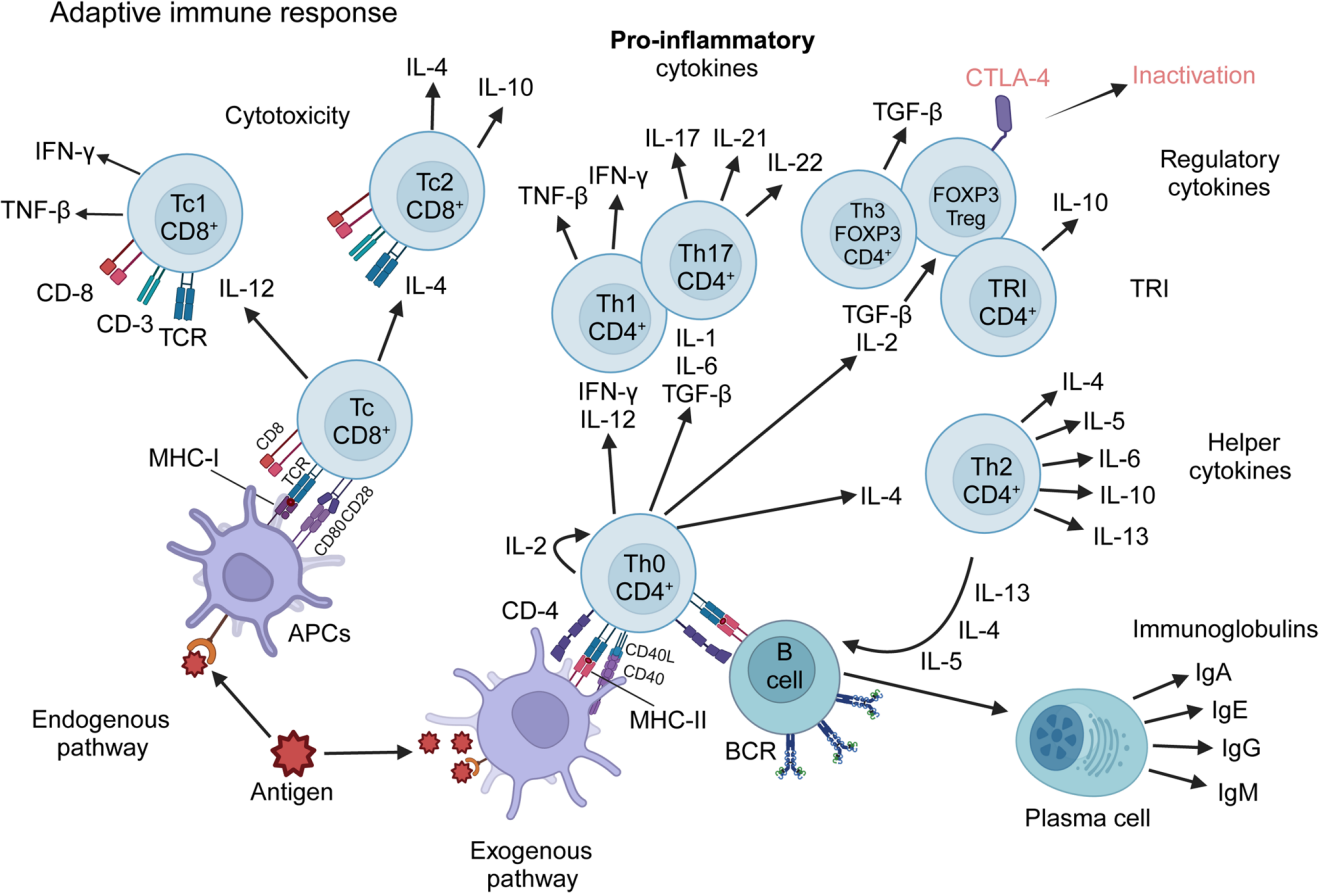
Schematic representation of the two major pathways in T cell differentiation. The Th and Tc populations, along with their respective subsets, are activated following antigen uptake and processing by APC, depicted here as a dendritic cell. The processed peptide is presented to the CD8^+^ population in the context of MHC-I, or to the CD4^+^ population in the context of MHC-II. This interaction triggers a cascade of lymphoproliferative and differentiative events, influenced by cytokines, which ultimately define the effector functions of these T cells. Figure created with BioRender.com

T cell differentiation is a highly regulated process essential for the development of distinct T cell subsets that orchestrate immune responses and maintain immune homeostasis [61–64]. Beginning in the thymus, naive T cells undergo positive and negative selection to establish a diverse, self-tolerant TCR repertoire [65, 66]. Upon encountering antigen-presenting cells (APCs) in peripheral tissues, naive T cells recognize antigen-MHC complexes through their TCR, receiving co-stimulatory signals that drive their differentiation into specialized subsets [67, 68]. The primary subsets include CD4^+^ helper T cells (Th cells), CD8^+^ cytotoxic T cells (Tc cells), and Tregs [42, 63] (Fig. 2). Th cells differentiate into distinct lineages, such as Th1, Th2, Th17, and T follicular helper (Tfh) cells, in response to specific cytokines, with each subset playing a unique role in immune regulation [69, 70]. Th1 cells, driven by IL-12 and the transcription factor T-bet, are crucial for cellular immunity against intracellular pathogens, while Th2 cells, induced by IL-4 and GATA3, are central to humoral immunity and allergic responses [71–73]. Th17 cells, regulated by IL-6, TGF-β, and RORγt, play a crucial role in mucosal immunity and inflammation. In parallel, Tfh cells are essential for supporting B cell activation and promoting antibody production. Both subsets contribute to the immune response through distinct but complementary mechanisms, influencing both local tissue immunity and systemic antibody-mediated defense [74–81]. Tc cells, influenced by IL-12 and T-bet, are key in targeting and eliminating infected or malignant cells. Tregs, which arise under the influence of TGF-β and are defined by FoxP3 expression, are vital for maintaining immune tolerance and preventing autoimmunity [82, 83]. The differentiation process is tightly controlled by the cytokine environment and the activation of lineage-specific transcription factors, such as T-bet, GATA3, RORγt, Bcl-6, and FOXP3, ensuring appropriate immune responses [59, 84–91]. Dysregulation of these pathways can lead to immune-related diseases, including autoimmunity, chronic inflammation, and cancer, underscoring the importance of understanding T cell differentiation for therapeutic development.

### T cell subsets and their roles in cancer

T cells are central to antitumor immunity, with distinct subsets playing diverse roles in cancer progression and immune surveillance [79, 92–95] (Table 1). CTLs mediate direct tumor cell killing through perforin- and granzyme-dependent mechanisms, while Th cells support antitumor responses by secreting cytokines that enhance CTL activity and modulate the tumor microenvironment [96–98]. However, Tregs, characterized by FOXP3 expression, suppress immune responses and contribute to tumor immune evasion by inhibiting effector T cell function [99, 100]. Unconventional T cell subsets, such as γδ T cells and NKT cells, also participate in antitumor immunity through MHC-independent pathways [101–103]. Although T cells have significant therapeutic potential, their efficacy in tumor control is often hindered by dysfunction arising from chronic antigen stimulation, immune checkpoint signaling, and metabolic constraints within the TME [93, 104–106]. A deeper understanding of T cell subset interactions and mechanisms of dysfunction is critical for advancing immunotherapies, including checkpoint blockade, adoptive T cell therapy, and tumor vaccines, to enhance durable antitumor immunity.

**Table 1:** Classification of T cells

Category		Subtype	Markers	Function	References
Conventional T cells		Th0	CD4, TCR, MHC-II	Enhance immune responses by secreting cytokines	[407]
		Th1	CD4, TNF-α, IFN-γ	Stimulates macrophages and enhances cell-mediated immunity	[408]
		Th2	CD4, IL-4, IL-5, IL-13	Promotes B cell differentiation and enhances defense against parasites	[409]
		Th17	CD4, IL-17, IL-22	Amplifies neutrophil response and combats extracellular pathogens	[410]
		Tfh	CD4, CXCR5, ICOS	Supports B cells in germinal centers to enhance antibody production	[78]
		Treg	CD4, FOXP3, IL-10	Regulates immune responses and preserves immune tolerance	[411]
		CTLs	CD8, TCR, MHC-I	Eliminates virus-infected and tumor cells through perforin and granzymes	[412]
Unconventional T cells		γδ T Cells	γδ TCR, CD3	Link innate and adaptive immunity while providing rapid responses to infections	[413, 414]
		NKT	TCR, CD1d	Detect lipid antigens and exhibit characteristics of both NK and T cells	[414, 415]
	MAIT		TCR, MR1	Recognize microbial metabolites and safeguard mucosal surfaces	[414, 416]

Th: CD4^+^ helper T cells; Tfh: Follicular helper cells; Treg: Regulatory T cells; CTLs: CD8^+^ cytotoxic T lymphocytes; NKT: Natural killer T cells; MAIT: Mucosal-associated invariant T cells

#### CD8⁺ cytotoxic T cells

**Fig. 3:**
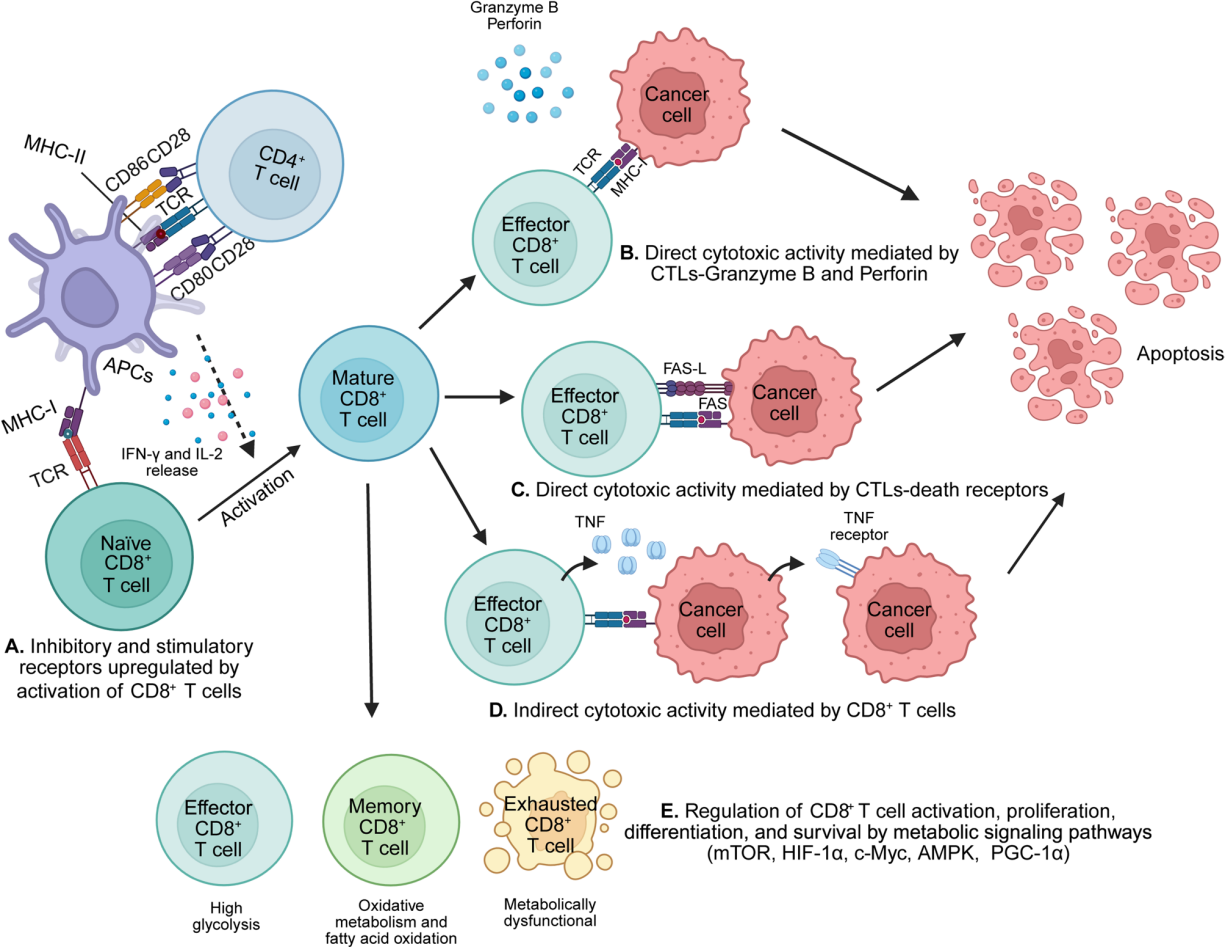
The role of CD8^+^ cytotoxic T cells in cancer. CTLs play a key role in cancer immunity by eliminating malignant and infected cells. Their activation is influenced by CD4^+^ T cells and APCs, which promote IL-2 secretion, while IFN-γ drives their maturation. Mature CD8^+^ T cells generate short-lived effector cells that kill tumor cells through direct cytotoxicity and indirect immune mechanisms. **(A)** Naïve CD8⁺ T cells recognize antigen presented by professional APCs, supported by CD4⁺ helper T cells, initiating their differentiation into functional cytotoxic T lymphocytes (CTLs). During activation, CD8⁺ T cells upregulate both stimulatory receptors (e.g., CD28, 4-1BB, OX40, ICOS) that promote proliferation and effector functions, and inhibitory receptors (e.g., PD-1, CTLA-4, LAG-3, TIM-3, TIGIT) that restrain excessive activation. Notably, PD-1 expression early after activation helps maintain immune homeostasis. The dynamic balance between these receptors determines the strength, duration, and outcome of the CD8⁺ T cell response, influencing effector activity, exhaustion, and memory development. **(B)** CTLs eliminate tumor cells through a direct cytotoxic mechanism involving perforin and granzyme. This process relies on cell-to-cell contact, which triggers the release of cytolytic enzymes, including granzyme B. Perforin forms pores in the target cell membrane, allowing granzyme B to enter and initiate apoptotic cell death. **(C)** Direct tumor cell killing occurs through the interaction between Fas ligand (Fas-L), expressed on CTLs, and its receptor Fas, present on cancer cells. This Fas/Fas-L binding triggers apoptosis in cancer cells via a caspase-dependent pathway. **(D)** Indirect CD8^+^ T cell-mediated killing: CTLs can induce indirect, or “bystander,” tumor cell death by secreting cytokines that exert their effects at a distance. For example, TNF-α secretion can activate apoptotic signaling in tumor cells expressing TNF receptors, contributing to immune-mediated tumor clearance. **(E)** Beyond antigen recognition, co-stimulation, and cytokines, metabolic reprogramming serves as a crucial fourth signal that controls CD8⁺ T cell function. Shifts in pathways like glycolysis, oxidative phosphorylation, and fatty acid metabolism support T cell proliferation, effector activity, and memory formation. Metabolic regulators such as mTOR and AMPK integrate these signals to shape T cell fate and function. Figure created with BioRender.com

CD8⁺ cytotoxic T cells are central to immune defense against infections and cancer, with their function intricately linked to metabolic programming [107, 108] (Fig. 3). Upon antigen recognition, naïve CD8⁺ T cells are activated—primarily by dendritic cells in secondary lymphoid organs—undergoing clonal expansion and differentiation into effector CTLs. This activation is accompanied by a profound metabolic shift toward increased glycolysis and oxidative phosphorylation to support biosynthetic demands, proliferation, and acquisition of effector functions such as cytokine production and cytolytic activity [109]. Effector CTLs eliminate tumor cells through two primary mechanisms: the perforin/granzyme pathway, wherein perforin facilitates granzyme B entry into target cells to induce apoptosis, and death receptor signaling, mediated via FasL–Fas and TRAIL–DR5 interactions that trigger caspase activation and cell death. In addition to direct cytotoxicity, CTLs secrete pro-inflammatory cytokines such as IFN-γ and TNF-α, which exert antiproliferative effects on tumor cells and promote immune remodeling of the TME. However, within the metabolically hostile TME, CD8⁺ T cells face a multitude of suppressive cues, including chronic antigen exposure, inhibitory checkpoint engagement (e.g., PD-1, LAG-3, TIM-3), immunosuppressive cytokines (e.g., TGF-β, IL-10), nutrient depletion, and hypoxia [110, 111]. These factors drive a dysfunctional or exhausted state marked by progressive loss of effector function, diminished cytokine production, impaired mitochondrial fitness, increased oxidative stress, and the acquisition of a distinct transcriptional and epigenetic landscape (e.g., TOX⁺, TCF1⁻ phenotype) [112, 113]. Modulating T cell metabolism—by enhancing mitochondrial biogenesis, targeting glycolytic flux, or disrupting metabolic checkpoints—represents a promising strategy to restore effector function and reinvigorate anti-tumor responses [114, 115]. Emerging evidence further suggests that systemic metabolic states, including dietary interventions and whole-body energy balance, influence CD8⁺ T cell differentiation and function, highlighting the need to consider organismal metabolism in immunotherapeutic design [116, 117]. A comprehensive understanding of how metabolism integrates with differentiation and effector fate decisions is essential for optimizing T cell-based therapies in cancer and chronic infection.

#### CD4^+^ helper T cells

**Fig. 4:**
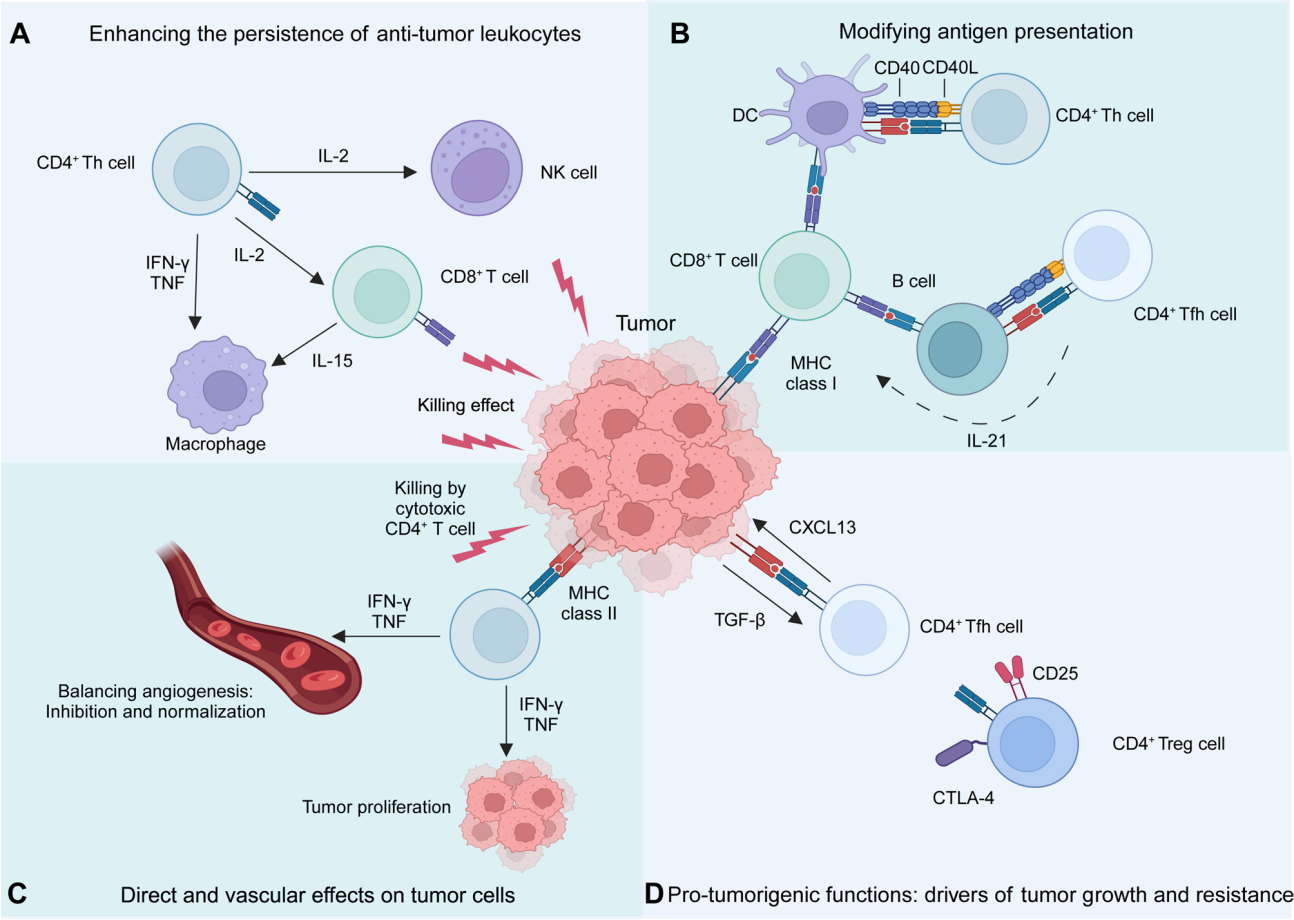
The role of CD4^+^ T cells in cancer. CD4^+^ T cell plasticity shapes innate and adaptive immunity within the TME, secondary lymphoid tissues, and tertiary lymphoid structures. **(A)** CD4^+^ T cells support the persistence and function of anti-tumor leukocytes, including NK cells, CD8^+^ T cells, and myeloid cell populations, through the secretion of cytokines such as IL-2, IFN-γ, and TNF. **(B)** In adjacent lymphoid tissues, they also influence antigen presentation by dendritic cells (DCs) and B cells through classical Th and Tfh cell functions, mediated by CD40L and IL-21. **(C)** In the TME, CD4^+^ T cells suppress tumor growth through cytokine production and cytotoxic activity, directly affecting tumor cells and modulating blood vessels. **(D)** CD4^+^ T cells play diverse and context-dependent roles in anti-tumor immunity, but they can also contribute to tumor progression through CD4^+^ Treg and Tfh cells. Key mechanisms include IL-2 consumption, suppression of antigen presentation via CTLA-4, and providing essential support for B cell lymphomas. The balance between their pro- and anti-tumor functions is a critical determinant of the immunogenicity of the TME, influencing disease progression and therapeutic outcomes. Figure created with BioRender.com

CD4^+^ T cells play a crucial role in cancer immunity beyond their traditional helper functions [92, 118] (Fig. 4). Although much of cancer immunotherapy has historically focused on CD8⁺ cytotoxic T lymphocytes, accumulating evidence highlights CD4⁺ T cells as central coordinators of both innate and adaptive immune responses, capable of exerting direct and indirect anti-tumor effects [119, 120]. Upon encountering tumor-associated antigens presented by MHC class II molecules on professional APCs, CD4⁺ T cells differentiate into specialized subsets—including Th1, Th2, Th17, Tfh, and Tregs —each with distinct transcriptional profiles, cytokine outputs, and functional consequences for the TME (Fig. 2). Among these, Th1 cells are most strongly associated with anti-tumor immunity. They produce IFN-γ and IL-2, license APCs, promote macrophage activation, and enhance CD8⁺ T cell priming and persistence [118, 121, 122]. In certain contexts, CD4⁺ T cells can also acquire cytotoxic potential via expression of perforin and granzyme B, directly killing MHC class II⁺ tumor cells. However, Tregs, characterized by FOXP3 and CD25 expression, suppress anti-tumor responses through both cytokine-dependent (e.g., IL-10, TGF-β, IL-35) and contact-mediated mechanisms, dampening the activity of effector T cells, dendritic cells, and NK cells [98, 123]. High Treg infiltration correlates with immune suppression and poor clinical outcomes in several tumor types.

The functional orientation of CD4⁺ T cells is shaped by cues from the TME, including chronic antigen exposure, cytokine gradients, and metabolic constraints such as glucose depletion and lactate accumulation. These factors drive cellular plasticity, potentially skewing effector subsets toward dysfunctional or immunosuppressive phenotypes [119, 120]. Notably, the formation of tertiary lymphoid structures within tumors facilitates local CD4⁺ T cell activation, B cell help via Tfh cells, and humoral immune responses.

Clinically, CD4^+^ T cells contribute to tumor immunity in diverse ways, including direct cytotoxicity against MHC class II-expressing tumor cells, supporting CD8^+^ T cell responses, and enhancing the efficacy of immune checkpoint blockade therapies [120, 124]. Their roles in cancer vaccines and adoptive T cell therapies are increasingly recognized, with evidence suggesting that CD4^+^ T cells may improve treatment outcomes [118, 125]. Future research aims to optimize therapeutic strategies by leveraging CD4^+^ T cell plasticity, functional specialization, and interactions within the TME to enhance anti-tumor immunity.

#### Regulatory T cells

**Fig. 5:**
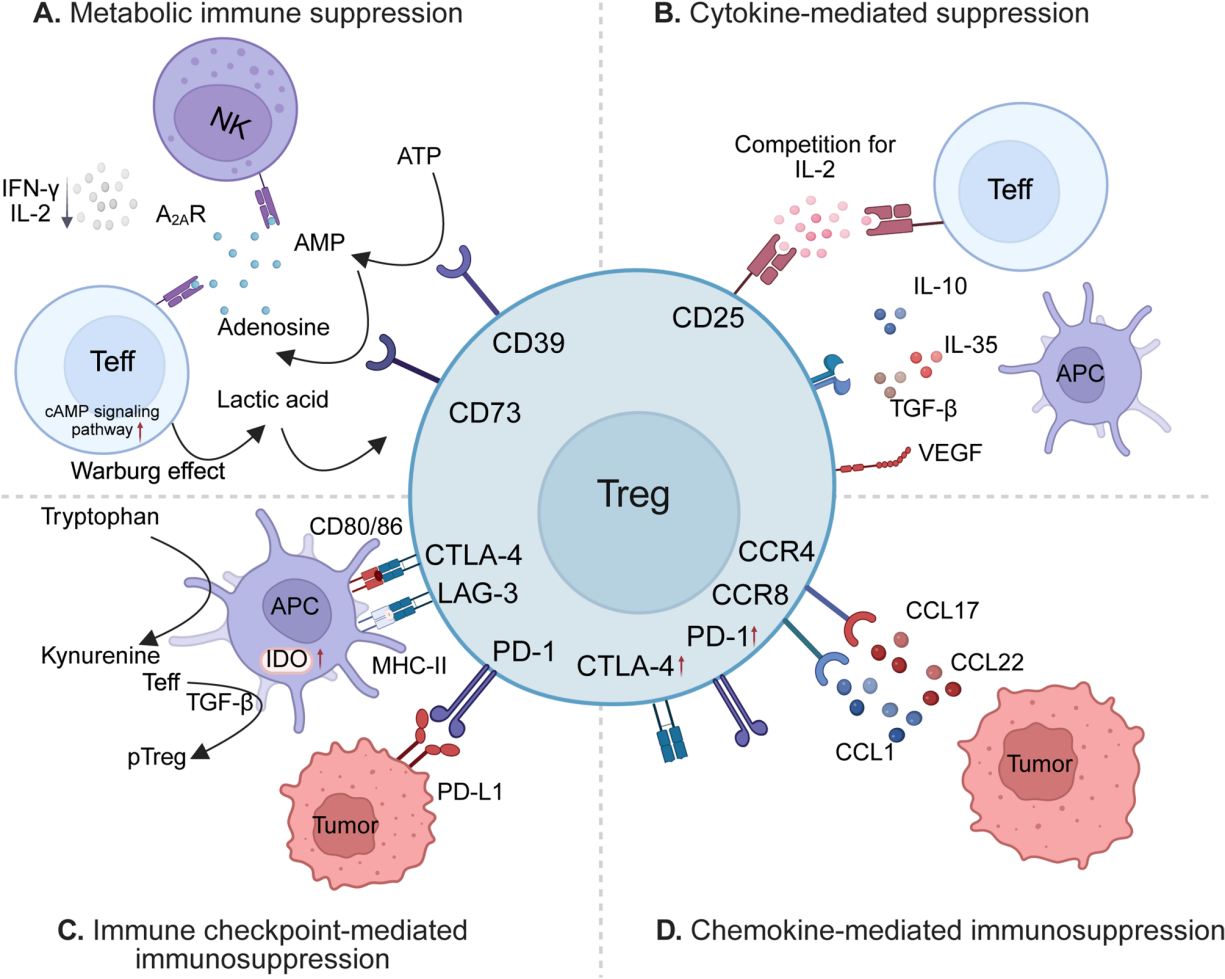
The role of Tregs in antitumor immunity regulation. **(A)** Tregs degrade ATP into adenosine through the enzymatic activity of CD39 and CD73, and adenosine suppresses the function of NK and effector T cells via A_2A_R signaling. Meanwhile, tumor cells, driven by the Warburg effect, accumulate lactate, which further promotes Treg generation. **(B)** Tregs suppress the immune response and promote tumorigenesis by competing with effector T cells for IL-2 via CD25 and secreting inhibitory cytokines, including IL-10, IL-35, TGF-β, and VEGF. **(C)** Tregs express surface markers such as CTLA-4, LAG-3, and PD-1, which interact with corresponding ligands on tumor cells and APCs to suppress effector T-cell activity. **(D)** Tregs migrate to the TME in response to chemokines such as CCL17, CCL22, and CCL1 by expressing corresponding receptors, including CCR4, CCR8, and PD-1. Figure created with BioRender.com

Regulatory T cells (Tregs) play a pivotal role in tumor immunity and have emerged as key mediators of resistance to cancer immunotherapy [126–128] (Fig. 5). In the TME, tumor-infiltrating Tregs often exhibit enhanced proliferation, which is considered a key mechanism enabling tumors to evade immune surveillance [17, 129, 130]. Their accumulation is associated with poor prognosis across a broad range of malignancies, including both solid tumors and hematologic cancers [99, 131–133]. Tregs suppress anti-tumor immunity through diverse and non-redundant mechanisms. These include the secretion of immunosuppressive cytokines such as IL-10, TGF-β, and IL-35; consumption of local IL-2 via high CD25 expression, leading to cytokine deprivation of effector T cells; and direct cytolytic activity mediated by granzyme/perforin pathways [130, 134–137]. Furthermore, Tregs downregulate costimulatory molecules (CD80/CD86) on APCs through CTLA-4–dependent trans-endocytosis, impairing CD8⁺ T cell priming and memory formation [138–141].

The functional fitness of intratumoral Tregs is supported by cues from the TME, including cytokines (e.g., IL-2, TGF-β), metabolic byproducts (e.g., adenosine, lactate, and kynurenine via IDO1 activity), and chemokines such as CCL22, CCL28, and CXCL12, which facilitate Treg recruitment through CCR4, CCR10, and CXCR4, respectively [134, 142–144]. In response to these signals, tumor-resident Tregs undergo transcriptional and epigenetic remodeling, often acquiring a tissue-adapted phenotype marked by high expression of immune checkpoints (e.g., CTLA-4, TIGIT, PD-1) and enhanced suppressive capacity. Epigenetically, this involves TET2/3-mediated demethylation at the FOXP3 locus and upregulation of transcriptional regulators such as Helios, IRF4, and BATF, which maintain lineage stability and suppressive function under inflammatory conditions. Given their central role in immune escape, Tregs have become a compelling therapeutic target for rebalancing the TME. Strategies aimed at reducing the Treg-to-effector T cell (Teff) ratio include depletion approaches using anti-CD25 antibodies or low-dose cyclophosphamide, as well as agents that block Treg-recruiting chemokines (e.g., CCR4 antagonists) or immune checkpoints like CTLA-4 and TIGIT [4, 145–150]. While these strategies have shown promise in preclinical and early clinical studies, their clinical translation is constrained by the risk of systemic immune dysregulation and autoimmunity resulting from broad Treg depletion [151–154]. To overcome this, emerging approaches focus on selectively modulating intratumoral Tregs without compromising systemic immune tolerance. These include antibody-drug conjugates targeting tumor-enriched Treg markers, metabolic disruption (e.g., adenosine or tryptophan pathway inhibitors), and engineered T cells or bispecific antibodies that preferentially deplete Tregs within the TME. Additionally, therapeutic strategies that reprogram rather than deplete Tregs—shifting them toward a less suppressive or even pro-inflammatory phenotype—are under investigation [141, 155, 156]. A deeper understanding of Treg heterogeneity, context-specific function, and plasticity will be essential to refine these approaches. High-resolution profiling of tumor-infiltrating Treg subsets, combined with spatial and functional analyses, holds promise for identifying actionable targets that can be exploited to enhance the efficacy of cancer immunotherapy while minimizing off-target effects.

#### Unconventional T cells

Unconventional T cells, including invariant natural killer T (iNKT) cells, MAIT cells, γδ T cells, and T cells restricted by monomorphic antigen-presenting molecules such as CD1 and MHC-related protein 1 (MR1), represent a unique class of lymphocytes that bridge innate and adaptive immunity [27, 101, 103, 157, 158]. Unlike conventional CD4⁺ and CD8⁺ αβ T cells that recognize peptide antigens presented by polymorphic MHC molecules, unconventional T cells respond to a diverse array of non-peptidic antigens—such as lipids, phosphoantigens, and microbial metabolites—presented by non-classical MHC class Ib-like molecules [159–161]. These cells exhibit potent anti-tumor properties through direct cytotoxicity and immune modulation, making them attractive targets for novel immunotherapeutic strategies. Their ability to recognize conserved antigens across different individuals also presents an advantage for “off-the-shelf” therapies.

Among these, γδ T cells are capable of recognizing stress-induced ligands or phosphoantigens presented by butyrophilin (BTN) and other non-classical MHC-like molecules in an MHC-independent manner. Subsets such as Vγ9Vδ2 and Vδ1⁺ T cells mediate anti-tumor responses via perforin/granzyme-dependent cytolysis and through engagement of death receptors (e.g., FasL, TRAIL), along with secretion of IFN-γ and TNF to promote effector cell recruitment and antigen presentation [160–162]. However, γδ T cells exhibit functional plasticity and can adopt pro-tumorigenic IL-17–producing phenotypes under TGF-β–rich or chronic inflammatory conditions, highlighting their dual roles in tumor immunity [163, 164].

MAIT cells, which recognize vitamin B2 (riboflavin) metabolites presented by MR1, are abundant in mucosal tissues and peripheral blood. Upon activation, they produce IFN-γ, granzyme B, and TNF and can contribute to tumor control [165, 166]. Nevertheless, in several cancers—including colorectal, hepatocellular, and hematologic malignancies—MAIT cells show impaired cytotoxicity and increased expression of exhaustion markers such as PD-1 and TIM-3, suggesting suppression by the TME [167, 168].

iNKT cells, restricted by CD1d and reactive to lipid antigens, are potent immune regulators with the capacity to rapidly secrete large quantities of IFN-γ, IL-4, and other cytokines [169]. iNKT cells promote anti-tumor immunity through both direct cytotoxic activity and transactivation of NK cells, dendritic cells, and CD8⁺ T cells. They also remodel the TME by reducing MDSCs and promoting M1 macrophage polarization [103, 170]. Conversely, certain subsets such as type II NKT cells may suppress anti-tumor immunity and facilitate tumor progression, underscoring the functional heterogeneity within the iNKT cell compartment [171–174].

Higher intratumoral infiltration of γδ T cells and other unconventional T cell subsets has been correlated with favorable prognosis in multiple malignancies [162, 175], yet their overall impact on tumor progression depends on local cues such as cytokine milieu, antigen availability, metabolic conditions, and chronic immune activation. Emerging high-dimensional profiling, including single-cell RNA-seq and spatial transcriptomics, has revealed previously unappreciated heterogeneity in unconventional T cell populations, indicating specialized, tissue- and tumor-specific roles [176, 177].

Given their rapid effector responses, MHC-independent antigen recognition, and potential for population-wide applicability, unconventional T cells are increasingly recognized as attractive candidates for immunotherapy. Clinical approaches under investigation include adoptive transfer of ex vivo–expanded γδ T cells, CD1d-restricted iNKT cell therapy, MR1 agonists, and engineered CAR–based therapies targeting these cell types [103, 157, 167, 170]. Importantly, their capacity to function in immunosuppressive TMEs and avoid classical mechanisms of immune evasion further supports their therapeutic potential. Continued mechanistic exploration of their biology and interactions with other immune and stromal components is essential to unlock their full value in next-generation cancer immunotherapy.

#### Tumor microenvironment and T cell dynamics

TME is characterized by a network of immunosuppressive mechanisms that hinder effective antitumor immunity [178–180]. Tregs, myeloid-derived suppressor cells (MDSCs), and tumor-associated macrophages (TAMs) contribute to an immunosuppressive milieu by secreting cytokines such as TGF-β and IL-10, which inhibit T cell activation [149, 181–183]. Additionally, metabolic constraints, including hypoxia and nutrient depletion, further impair T cell functionality [114, 184, 185]. Tumor cells themselves exploit immune checkpoint pathways, such as PD-1/PD-L1 and CTLA-4 signaling, to evade immune recognition, making it difficult for T cells to mount an effective response [6, 186, 187]. These suppressive mechanisms collectively contribute to the persistence and progression of tumors despite the presence of tumor-infiltrating lymphocytes.

A major consequence of the immunosuppressive TME is the induction of T cell exhaustion, a dysfunctional state marked by sustained expression of inhibitory receptors, metabolic insufficiency, and reduced cytokine production [188, 189]. Exhausted T cells arise due to chronic antigen exposure, leading to progressive loss of proliferative and cytotoxic capabilities [13, 190]. Recent studies have identified subsets of exhausted T cells, including progenitor exhausted T cells, which retain some functional potential and may be reinvigorated through immune checkpoint blockade [191–193]. However, the effectiveness of ICB is often limited by the presence of deeply exhausted T cell populations that fail to recover full effector function [188, 194, 195]. Understanding the molecular drivers of T cell exhaustion is critical for designing therapies that can reverse dysfunction and sustain long-term antitumor responses.

Beyond T cells, the TME is composed of a dynamic network of immune and stromal cells that engage in complex crosstalk, influencing tumor progression and immune responses [196–198]. Cancer-associated fibroblasts (CAFs) remodel the extracellular matrix and secrete immunosuppressive factors that limit T cell infiltration [199–201]. Meanwhile, DCs within the TME exhibit impaired antigen presentation, reducing the priming and activation of tumor-reactive T cells [202–205]. Additionally, interactions between T cells and myeloid cells, including MDSCs and TAMs, further promote immune suppression through the release of arginase and reactive oxygen species [206–208]. Understanding these cellular interactions is essential for developing combination therapies that not only restore T cell function but also reprogram the TME to support durable immune responses against cancer.

#### Molecular pathways regulating T cell function

T cells are crucial mediators of the adaptive immune response, recognizing antigenic peptides presented by MHC molecules. The engagement of the TCR with antigen-MHC complexes triggers a cascade of intracellular signaling events essential for T cell activation, differentiation, and immune response modulation [49, 209]​. These molecular pathways, orchestrated by kinases, phosphatases, adaptor proteins, and secondary messengers, govern T cell function in health and disease. Dysregulation of these pathways can lead to immune tolerance, autoimmunity, or impaired immune responses [210, 211].

**Fig. 6:**
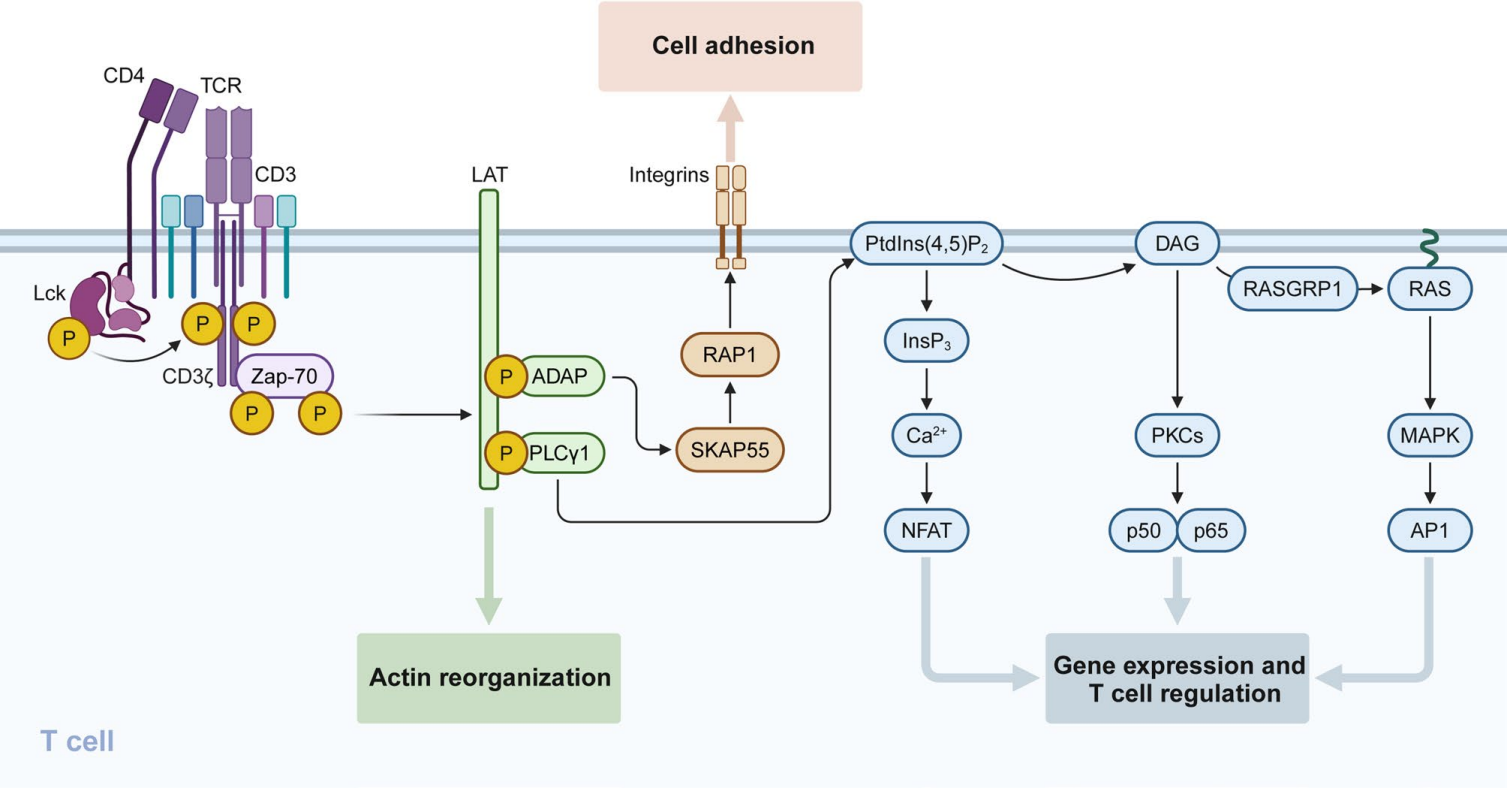
Schematic diagram of TCR signaling network. TCR signaling is initiated by the recognition of peptide–MHC complexes, leading to the recruitment of Lck, which phosphorylates ITAMs on the CD3γ, CD3δ, CD3ɛ, and ζ-chains. This enables Zap-70 recruitment, phosphorylation, and activation. Activated ZAP70 phosphorylates LAT, assembling the LAT signalosome, which includes PLCγ1, GRB2, GADS, SLP76, ADAP, ITK, NCK1, and VAV1. This complex propagates signaling through the Ca²⁺, MAPK, and NF-κB pathways, driving transcriptional activation, T cell proliferation, and differentiation. Additionally, TCR signaling regulates actin reorganization and integrin activation, enhancing immune synapse formation. SKAP55, SRC kinase-associated phosphoprotein of 55 kDa; PtdIns [4, 5]P2, phosphatidylinositol-4,5-bisphosphate; InsP3, inositol-1,4,5-trisphosphate; NFAT, nuclear factor of activated T cells; DAG, diacylglycerol; PKC, protein kinase C; RASGRP1, RAS guanyl-releasing protein 1; AP1, activator protein 1. Figure created with BioRender.com

TCR activation begins with phosphorylation of immunoreceptor tyrosine-based activation motifs within the CD3ζ chain by Lck, a Src family kinase [212] (Fig. 6). This recruits and activates ZAP-70, which phosphorylates adaptor proteins such as linker for activation of T cells (LAT) and SH2 domain-containing leukocyte protein of 76 kDa (SLP-76), forming a multi-protein signaling complex​ [213–215]. These early events enable the activation of multiple distal signaling pathways, including the Ca²⁺-calcineurin-NFAT pathway, PKCθ-IKK-NFκB axis, RAS-MAPK cascade, and the mTOR signaling network [216–221]​. These pathways integrate signals for T cell proliferation, cytokine production, and metabolic adaptation to immune responses.

To maintain immune homeostasis, inhibitory pathways tightly regulate TCR signaling. The phosphatases SHP-1 and SHP-2 dephosphorylate key signaling molecules, limiting TCR activation [222, 223]. Additionally, transmembrane adaptor proteins such as PAG/CBP recruit C-terminal Src kinase (CSK) to suppress Lck activity in resting T cells [224–226]​. Diacylglycerol kinases (DGKs) further restrain T cell activation by metabolizing DAG, a key secondary messenger in the RAS-ERK1/2 pathway [227, 228]. Dysregulation of these inhibitory pathways can lead to T cell hyperactivation, contributing to autoimmunity or immune exhaustion. In addition to TCR signaling, co-stimulatory molecules such as CD28 are essential for preventing T cell anergy and promoting full activation. CD28 engagement amplifies PI3K-AKT-mTOR signaling, a pathway critical for metabolic reprogramming of activated T cells [229, 230]​. The mTORC1 complex facilitates Th1 and Th17 differentiation, while mTORC2 supports Th2 and Treg lineage commitment [221, 231]. Fine-tuning of mTOR signaling ensures balanced immune responses and prevents metabolic exhaustion of effector T cells.

Understanding the molecular pathways governing T cell function has profound implications for immunotherapy. Strategies targeting key signaling nodes, such as immune checkpoint inhibitors (e.g., PD-1 blockade), metabolic reprogramming (mTOR inhibition), and engineered TCR or CAR-T cell therapies, leverage these pathways to enhance anti-tumor immunity while minimizing autoimmunity​. Further insights into these regulatory circuits will continue to refine immunotherapeutic approaches and improve clinical outcomes in immune-related diseases.

### Therapeutic advances in T cell-based cancer therapies

Recent therapeutic advances in T cell-based cancer therapies have significantly improved clinical outcomes, particularly with the development of ICIs, T cell engagers (TCEs), and ACT [20, 27, 106, 232–234]. In particular, CAR T cells and TCR-engineered T cells have demonstrated remarkable efficacy in hematologic malignancies, with CD19-directed CAR T cells achieving durable remissions in B cell malignancies and BCMA-targeted therapies transforming the treatment landscape of multiple myeloma [27, 235, 236]. Several CAR T cell therapies, including those targeting CD19 and BCMA, have received regulatory approval from the Food and Drug Administration (FDA) and National Medical Products Administration of China (NMPA), underscoring their clinical impact [237–240] (Table 2). Innovations such as dual-targeting CARs, armored T cells, and next-generation gene editing techniques aim to enhance persistence, mitigate immune evasion, and overcome antigen loss [28, 241–243]. Additionally, allogeneic and “off-the-shelf” T cell therapies are being explored to address manufacturing challenges and broaden accessibility [244, 245]. However, toxicities such as CRS and immune effector cell-associated neurotoxicity syndrome (ICANS) remain critical concerns, necessitating refined approaches for safer and more effective applications [242, 246, 247]. Continued advancements in T cell engineering, tumor microenvironment modulation, and combination strategies with immune checkpoint inhibitors hold promise for expanding the efficacy of T cell-based therapies across a wider range of hematologic and solid malignancies.

**Table 2:** T cell-based therapies that have received regulatory approval for commercialization in the U.S. Or China

Therapy type	Classification	Product name	Target	Type of cancer targeted against	Brand	Approval date
ICI	mAbs	Pembrolizumab	PD-1	Various cancers	Keytruda	2014 FDA
		Nivolumab	PD-1	Various cancers	Opdivo	2014 FDA
		Cemiplimab	PD-1	Various cancers	Libtayo	2018 FDA
		Dostarlimab	PD-1	Various cancers	Jemperli	2021 FDA
		Atezolizumab	PD-L1	Various cancers	Tecentriq	2016 FDA
		Avelumab	PD-L1	Various cancers	Bavencio	2016 FDA
		Durvalumab	PD-L1	Various cancers	Imfinzi	2016 FDA
		Cosibelimab-ipdl	PD-L1	metastatic or locally advanced CSCC	Unloxcyt	2024 FDA
		Ipilimumab	CTLA-4	Various cancers	Yervoy	2010 FDA
		Tremelimumab	CTLA-4	HCC	Imjudo	2022 FDA
	Combination	Relatlimab + Nivolumab	Lag-3 + PD-1	Metastatic melanoma	Opdual + Opdivo	2022 FDA
		Nivolumab + Hyaluronidase-nvhy	PD-1	Multiple adult solid cancer types	Opdivo + Qvantig	2024 FDA
		Atezolizumab + Hyaluronidase-tqjs	PD-L1	Multiple cancer types	Tecentri + Hybreza	2024 FDA
	bsAbs	Cadonilimab	PD-1 x CTLA-4	Metastatic cervical cancer	Ak104	2022 NMPA
TCE	bsAbs	Blinatumomab	CD3 x CD19	r/r ALL	Blincyto	2014 FDA
		Mosunetuzumab-axgb	CD3 x CD20	FL	Lunsumio	2022 FDA
		Teclistamab-cqyv	CD3 x BCMA	r/r MM	Tecvayli	2022 FDA
		Elranatamab	CD3 x BCMA	r/r MM	Elrexfio	2023 FDA
		Tarlatamab-dlle	CD3 x DLL3	Extensive stage SCLC	Imdelltra	2024 FDA
		Epcoritamab-bysp	CD3 x CD20	r/r FL	Epkinly	2024 FDA
	TCR-like antibody	Tebentafusp-tebn	CD3 x gp100 peptide-HLA	Uveal melanoma	Kimmtrak	2022 FDA
		Afamitresgene autoleucel	MAGE-A4	Certain patients with metastatic SSC	Tecelra	2024 FDA
ACT	CAR-T	Axicabtagene ciloleucel	CD19	NHL, DLBCL	Yescarta	2017 FDA
		Tisagenlecleucel	CD19	ALL, DLBCL	Kymriah	2017 FDA
		Brexucabtagene autoleucel	CD19	ALL, MCL	Tecartus	2020 FDA
		Lisocabtagene maraleucel	CD19	DLBCL	Breyanzi	2021 FDA
		Relmacabtagene autoleucel	CD19	DLBCL	Relma-cel	2021 NMPA
		Obecabtagene autoleucel	CD19	r/r ALL	Aucatzyl	2024 FDA
		Idecabtagene vicleucel	BCMA	MM	Abecma	2021 FDA
		Ciltacabtagene autoleucel	BCMA	MM	Carvykti	2022 FDA
		Equecabtagene autoleucel	BCMA	r/r MM	Fucaso	2023 NMPA
		Zevorcabtagene autoleucel	BCMA	r/r MM	Zevo-cel	2024 NMPA

ICI: immune checkpoint inhibitor, TCE: T cell engager, ACT: adoptive cell therapy, HCC: hepatocellular carcinoma, mAbs: monoclonal antibodies, bsAbs: bispecific antibodies, CSCC: cutaneous squamous cell carcinoma, r/r ALL: relapsed/refractory B cell precursor acute lymphoblastic leukemia, r/r FL: relapsed/refractory follicular lymphoma, r/r MM: relapsed/refractory multiple myeloma, SCLC: small cell lung cancer, SSC: synovial sarcoma, NHL: non-Hodgkin’s lymphoma, DLBCL: diffuse large B-cell lymphoma, MCL: mantle cell lymphoma, FDA: Food and Drug Administration, NMPA: National Medical Products Administration of China

#### Immune checkpoint inhibitors (ICIs)

Modulating co-inhibitory and co-stimulatory molecules on T cells has emerged as a powerful strategy in cancer immunotherapy, allowing for precise regulation of immune activation to enhance therapeutic efficacy [248, 249]. Immune checkpoint molecules, a subset of inhibitory receptors expressed on immune cells, play a crucial role in maintaining self-tolerance by dampening excessive immune responses [250, 251]. Among them, cytotoxic T-lymphocyte-associated protein 4 (CTLA-4) and programmed death-1 (PD-1) have been the most clinically successful targets [24, 252]. These checkpoints suppress T cell activation upon ligand engagement, allowing tumors to evade immune surveillance [253–255]. Blocking these inhibitory pathways with ICIs restores T cell function, reinvigorates antitumor immunity, and enhances immune-mediated tumor clearance.

The clinical development of ICIs has transformed cancer treatment, beginning with the approval of ipilimumab, a mAb targeting CTLA-4, over a decade ago. Since then, eight ICIs targeting PD-1/PD-L1, as well as another CTLA-4 inhibitor, tremelimumab, have been approved for multiple malignancies (Table 2). Notably, a large number of clinical trials are currently evaluating anti-PD-1/PD-L1 mAbs, either as monotherapy or in combination with other therapeutic modalities (Table 3). Beyond PD-1/PD-L1 and CTLA-4, additional immune checkpoint pathways, including lymphocyte-activation gene 3 (LAG-3), T cell immunoreceptor with Ig and ITIM domains (TIGIT), T cell immunoglobulin and mucin-domain containing-3 (Tim-3), CD96, B and T lymphocyte attenuator (BTLA), V-domain Ig suppressor of T cell activation (VISTA), and B7-H3, have been identified as potential therapeutic targets [24, 256–260]. The anti-LAG-3 mAb, telatlimab, was the first to receive FDA approval for the treatment of metastatic melanoma, where it is used in combination with an anti-PD-1 mAb. Furthermore, several checkpoint inhibitors targeting LAG-3, TIGIT, vascular endothelial growth factor (VEGF) and Tim-3 are in late-stage clinical development [261–264].

**Table 3:** Clinical studies of T cell-based therapies (selected)

Therapy type	Classification	Product name	Phase	Target	Type of cancer targeted against	Clinical design	Clinical response	References
ICI	mAbs	Tiragolumab	III (NCT04294810)	TIGIT	NSCLC	Tiragoluma + Atezolizumab	Completed.	Not found.
		Ociperlimab	III (NCT04746924)	TIGIT	NSCLC	Ociperlima + Tislelizumab	Completed.	Not found.
		MBG453	III (NCT04266301)	Tim-3	MS	MBG453 + Azacitidine	ORR: 56.9%.	[261]
		Fianlimab	III (NCT05608291)	Lag-3	Melanoma	Fianlimab + Cemiplima vs. Pembrolizumab	Recruiting.	Not found.
		Feladilimab	II/III (NCT04428333)	ICOS	Recurrent/metastatic head and neck SCC	Feladilimab + Pembrolizumab + 5FU-Platinum Chemotherapy	Terminated.	Not found.
	bsAbs	Tebotelimab	II/III (NCT04082364)	PD-1 × Lag-3	GC	Margetuximab + Retifanlimab + Tebotelimab + Chemotherapy	ORR: 47%.	[262]
		Lomvastomig	II (NCT04785820)	PD-1 x Tim-3	Advanced/metastatic ESCC	Lomvastomig + Tobemstomig	Completed.	Not found.
		Rilvegostomig	I/II (NCT04995523)	PD-1 x TIGIT	NSCLC	Rilvegostomig	Recruiting.	Not found.
		XmAb23104	I (NCT05695898)	PD-1 x ICOS	Advanced/metastatic melanoma	XmAb23104 (PD1 X ICOS) vs. XmAb22841 (CTLA-4 X LAG3)	Active.	Not found.
		Ivonescimab	III (NCT05499390)	PD-1 x VEGF	Advanced NSCLC	Ivonescimab vs. Pembrolizumab	mPFS: Ivonescimab vs. pembrolizumab (11.1 vs. 5.8 months).	[263]
		PM8002	II/III (NCT05756972)	PD-1 x VEGF	NSCLC	PM8002 + Chemotherapy	Active.	Not found.
		KN046	III (NCT04474119)	PD-L1 x CTLA- 4	NSCLC	KN046 + Platinum-containing Chemotherapy vs. Placebo + Platinum-containing Chemotherapy	Unknown status.	Not found.
		FS118	I/II (NCT03440437)	PD-L1 × Lag-3	Advanced malignancies	FS118 or FS118 + Paclitaxel	ORR: 46.5%.	[264]
		Acasunlimab	I/II (NCT03917381)	PD-L1 × 4-1BB	Malignant solid tumors	Casunlimab or casunlimab + docetaxel + pembrolizumab	ORR: 65.6%.	[275]
		PRS-344/S095012	I/II (NCT05159388)	PD-L1 × 4-1BB	Advanced and/or metastatic solid tumors	PRS-344/S095012	Active.	Not found.
		Bintrafusp alfa (M7824)	III (NCT03631706)	PD-L1 x TGF-βRII	PD-L1-high advanced NSCLC	Bintrafusp alfa vs. pembrolizumab	mPFS: Bintrafusp alfa vs. pembrolizumab (7.0 vs. 11.1 months); mOS: 21.1 vs. 22.1 months.	[417]
		SHR-1701	III (NCT05132413)	PD-L1 x TGF-βRII	Advanced/metastatic NSCLC	SHR-1701 + Pemetrexed Disodium + Cisplatin/carboplatin + Bevacizumab	Unknown status.	Not found.
		XmAb22841	I (NCT05695898)	CTLA-4 × Lag-3	Metastatic melanoma	XmAb23104 (PD1 X ICOS) + XmAb22841 (CTLA-4 X LAG3)	Active.	Not found.
TCE	CD3 x TAA	Epcoritamab	III (NCT04628494)	CD3 x CD20	DLBCL	Epcoritamab	Active.	Not found.
		Glofitamab	III (NCT04408638)	CD3 x CD20	DLBCL	Glofitamab + Gemcitabine + Oxaliplatin vs. Rituximab + Gemcitabine + Oxaliplatin	mOS: Glofit-GemOx vs. R-GemOx (25.5 vs. 12.9 months).	[282]
		Elranatamab	III (NCT05317416)	CD3 x BCMA	r/r MM	Elranatamab vs. Lenalidomide	Recruiting.	Not found.
		Teclistamab	III (NCT05083169)	CD3 x BCMA	r/r MM	Tec-Dara vs. DPd/DVd	Active.	Not found.
		Linvoseltamab	III (NCT05730036)	CD3 x BCMA	r/r MM	Linvoseltamab vs. EPd	Recruiting.	Not found.
		Talquetamab	III (NCT05455320)	CD3 x GPRC5D	r/r MM	Tal-DP or Tal-D vs. DPd	Recruiting.	Not found.
		Catumaxomab	III (NCT04222114)	CD3 x EpCAM	Advanced GC with peritoneal metastasis	Catumaxomab	Unknown status.	Not found.
		Tarlatamab	III (NCT05740566)	CD3 x DLL3	Relapsed SCLC	Tarlatamab with standard of care chemotherapy	ORR: 40%.	[283]
		REGN4336	I/II (NCT05125016)	CD3 x PSMA	Advanced prostate cancer	REGN4336 alone or REGN4336 + Cemiplimab or REGN5678 (a PSMAxCD28 Bispecific Antibody)	Recruiting.	Not found.
		CC-1	I/II (NCT04496674)	CD3 x PSMA	SCCL	CC-1	Terminated.	Not found.
		REGN4018	I/II (NCT03564340)	CD3 x MUC16	Recurrent advanced OC	REGN4018 alone or in combination with Cemiplimab	Recruiting.	Not found.
		EGFR BATs	I/II (NCT03269526)	CD3 x EGFR	Advanced or metastatic PC	EGFRBi armed activated T cells (EGFR BATs)	Active.	Not found.
		Cibisatamab	I (NCT03866239)	CD3 x CEA	Metastatic CC	Cibisatamab + Atezolizumab	Terminated.	Not found.
		Runimotamab	I (NCT03448042)	CD3 x HER2	Advanced or metastatic HER2-expressing cancers	Runimotamab or Runimotamab + Trastuzumab	Active.	Not found.
		AMG 596	I (NCT03296696)	CD3 x EGFRvIII	EGFRvIII positive GBM or malignant glioma	AMG 596 or AMG 596 + AMG 404	Terminated.	Not found.
		GEM3PSCA	I (NCT03927573)	CD3 x PSCA	Cancers with positive PSCA	GEM3PSCA	Terminated.	Not found.
		ERY974	I (NCT05022927)	CD3 x GPC3	HCC	ERY974 + Tocilicumab + Atezolizumab + Bevacizumab	Active.	[418]
	CD28 x TAA	REGN5668	I/II (NCT04590326)	CD28 x MUC16	OC	REGN5668 + Cemiplimab or REGN4018 (with or without sarilumab)	Recruiting.	Not found.
		REGN5678	I/II (NCT03972657)	CD28 x PSMA	mCRPC and ccRCC	REGN5678 or REGN5678 + Cemiplimab	Recruiting.	Not found.
		REGN7075	I/II (NCT04626635)	CD28 x EGFR	Advanced solid tumors	REGN7075 + Cemiplimab + Platinum-based doublet chemotherapy	Recruiting.	Not found.
	4-1BB x TAA	Acasunlimab	II (NCT05117242)	4-1BB x PD-L1	NSCLC	Acasunlimab or Acasunlimab + Pembrolizumab	Active.	Not found.
		Cinrebafusp alfa	II (NCT05190445)	4-1BB x HER2	HER2-positive GC	Cinrebafusp alfa + Ramucirumab + Paclitaxel or Cinrebafusp alfa + Tucatinib	Active.	Not found.
		HLX35	I (NCT05360381)	4-1BB x EGFR	Advanced or metastatic solid tumors	HLX35	Active.	Not found.
		CB307	I (NCT04839991)	4-1BB x PSMA	Advanced and/​or metastatic PSMA-positive tumours	CB307	Recruiting.	Not found.
		RO7122290	I/II (NCT04826003)	4-1BB x FAP	Metastatic CC	RO7122290 + Cibisatamab + Obinutuzumab	Completed.	Not found.
		BT7480	I/II (NCT05163041)	4-1BB x Nectin-4	Advanced solid tumor	BT7480 or BT7480 + Nivolumab	Active.	Not found.
ACT	CAR-T	CD19 CAR-T	II/III (NCT04257175)	CD19	r/r AML	CD19 targeted CAR-T cells	CR: 66.7%.	[304]
		CD19 CAR-T	III (NCT05020392)	CD19	DLBCL	CD19 targeted CAR-T cells	Recruiting.	Not found.
		CD19 CAR-T	II/III (NCT03937544)	CD19	r/r ALL	CD19 targeted CAR-T cells + CTX + FAMP	Unknown status.	Not found.
		BCMA CAR-T (Bb2121)	I (NCT02658929)	BCMA	r/r MM	BCMA targeted CAR-T cells + CTX + FAMP	ORR: 75.8%; CR: 38.7%; mPFS: 18.1 months.	[305]
		BCMA CAR-T (LCAR-B38M)	I/II (NCT03090659)	BCMA	r/r MM	BCMA targeted CAR-T cells + CTX	ORR: 87.8%; CR: 73%; MRD negativity: 67.6%; 5-year PFS: 21%; 5-year OS: 49.1%.	[306]
		BCMA CAR-T (JNJ-4528)	I/II (NCT03548207)	BCMA	r/r MM	BCMA targeted CAR-T cells + CTX + FAMP	ORR: 97.9%; sCR: 82.5%; 27-month PFS: 54.9%; 27-month OS: 70.4%.	[307]
		BCMA CAR-T (CART-BCMA)	I (NCT02546167)	BCMA	r/r MM	None or CTX + BCMA targeted CAR-T cells	ORR: 48%; CR: 8%; VGPR: 20%; PR: 20%.	[308]
		BCMA CAR-T (MCARH171)	I (NCT03070327)	BCMA	r/r MM	BCMA targeted CAR-T cells + CTX + FAMP	ORR:64%.	[309]
		GPRC5D (MCARH109)	I (NCT04555551)	GPRC5D	r/r MM	GPRC5D-targeted CAR-T cells	ORR: 71%; CR: 35%; MRD negativity: 47%.	[312]
		GPRC5D (BMS-986393)	I (NCT04674813)	GPRC5D	r/r MM	GPRC5D-targeted CAR-T cells	Patients with assessable efficacy: ORR: 86%; CR: 38%; patients refractory to prior BCMA-directed therapy: ORR: 85%; CR: 46%.	[311]
		GPRC5D (OriCAR-017)	I (NCT05016778)	GPRC5D	r/r MM	GPRC5D-targeted CAR-T cells	ORR: 100%; sCR: 60%.	[313]
		B7H3 CAR-T	I/II (NCT05211557)	B7H3(CD276)	Recurrent malignant OC	fhB7H3 CAR-T cells	Recruiting.	Not found.
		B7H3 CAR-T	I/II (NCT05143151)	B7H3(CD276)	Advanced PC	CD276 CAR-T cells	Unknown status.	Not found.
		MESO CAR-T	I/II (NCT03916679)	Mesothelin	OC	MESO CAR-T Cells	Unknown status.	Not found.
		ALPP CAR-T	I/II (NCT04627740)	Alkaline phosphatase	EC	ALPP CAR-T cells	Recruiting.	Not found.
		LGR5 CAR-T (CNA3103)	I/II (NCT05759728)	LGR5	Metastatic CC	LGR5 CAR-T cells	Recruiting.	Not found.
		Claudin18.2 CAR-T (CT041)	I/II (NCT04404595)	Claudin18.2	GC/PC	Claudin18.2 CAR-T cells	Active.	Not found.
		ROR1 CAR-T (RD14-01)	I/II (NCT05748938)	ROR1	Solid tumors	ROR1 CAR-T cells	Unknown status.	Not found.
		CEA CAR-T	I/II (NCT04348643)	CEA	Advanced solid tumors	CEA CAR-T cells	Unknown status.	Not found.
		HLA-G CAR-T (IVS-3001)	I/II (NCT05672459)	HLA-G	Advanced solid tumors	HLA-G CAR-T cells	Recruiting.	Not found.
		PSCA CAR-T (BPX-601)	I/II (NCT02744287)	PSCA	Metastatic PC or mCRPC	PSCA CAR-T cells + Rimiducid	SD: 61.5%; PD: 30.8%.	[419]
		HypoSti. HER2 CAR-T	I/II (NCT05681650)	HER2	HER2 positive advanced solid tumors	HER2 CAR-T cells	Recruiting.	Not found.
		CLDN6 CAR-T	I (NCT04503278)	Claudin 6	Advanced solid tumors	CLDN6 CAR-T cells	ORR: 57%.	[420]
		GD-2 CAR-T	I/II (NCT03373097)	GD-2	High Risk and/or r/r neuroblastoma	GD-2 CAR-T cells	ORR: 63%; CR: 33.3%; PR: 29.6%.	[421]
		MUC1 CAR-T	I/II (NCT03633773)	MUC1	ICC	MUC1 CAR-T cells	Unknown status.	Not found.
		GPC3 CAR-T (BOXR1030)	I/II (NCT05120271)	Glypican-3	GPC3 positive advanced solid tumors	GPC3 CAR-T cells	Recruiting.	Not found.
	Bispecific CAR-T	bi-4SCAR CD19/20 T cells	I/II (NCT04186520)	CD19/CD20	B cell malignancies	CD19/CD20 CAR-T cells	CAR-20/19-T cells may have efficacy in CD19- patient populations.	[315]
		bi-4SCAR CD19/22 T cells	I/II (NCT05432882)	CD19/CD22	B cell malignancies	CD19/CD22 CAR-T cells	Recruiting.	Not found.
		bi-4SCAR CD19/70 T cells	I/II (NCT05436496)	CD19/CD70	B cell malignancies	CD19/CD70CAR-T cells	Recruiting.	Not found.
		bi-4SCAR CD19/79b T cells	I/II (NCT05436509)	CD19/CD79b	B cell malignancies	CD19/CD79b CAR-T cells	Recruiting.	Not found.
		BC19 CAR-T cells	I/II (ChiCTR2000033567)	CD19/BCMA	r/r MM	CD19/BCMA CAR-T cells	ORR: 92%; mPFS: 19.7 months; mOS: 19.7 months.	[316]
		BM38 CAR-T cells	I (ChiCTR1800018143)	CD38/BCMA	r/r MM	CTX + FAMP + CD38/BCMA CAR-T cells	ORR: 87%; CR: 52%.	[317]
		BM38 CAR-T cells	I (ChiCTR1900026286)	CD38/BCMA	r/r MM	CTX + FAMP + CD38/BCMA CAR-T cells	ORR: 88%; CR: 81%.	[318]
		bi-4SCAR CS1/BCMA T cells	I/II (NCT04662099)	CS1/BCMA	r/r MM	CTX + FAMP + CS1/BCMA CAR-T cells	ORR: 81%; sCR: 38%.	[319]
		bi-4SCAR GD2/CD70 T cells	I/II (NCT05438368)	GD2/CD70	Cancer disease	GD2/CD70 CAR-T cells	Recruiting.	Not found.
		bi-4SCAR GD2/PSMA T cells	I/II (NCT05437315)	GD2/PSMA	Cancer disease	GD2/PSMA CAR-T cells	Recruiting.	Not found.
		bi-4SCAR PSMA/CD70 T cells	I/II (NCT05437341)	PSMA/CD70	Cancer disease	PSMA/CD70 CAR-T cells	Recruiting.	Not found.
		bi-4SCAR VEGFR1/PD- L1 T cells	I (NCT05477927)	VEGFR1/PD- L1	Malignant peritoneal effusion	VEGFR1/PD- L1 CAR-T cells	Recruiting.	Not found.
		bi-4SCAR EGFR/B7H3 T cells	I (NCT05341492)	EGFR/B7H3	EGFR/ B7H3-positive advanced solid tumors (lung cancer and TNBC)	EGFR/B7H3 CAR-T cells	Recruiting.	Not found.
		bi-4SCAR HER2/PD-L1 T cells	I (NCT04684459)	HER2/PD-L1	HER2-positive solid tumors	HER2/PD-L1 CAR-T cells	Recruiting.	[320]
	TCR-T	NY-ESO-1-LAGE-1 TCR-engineered T cells	I/II (NCT01352286)	NY-ESO-1	NY-ESO-1 positive MM	NY-ESO-1-specific TCR-engineered T cells after ASCT.	CR: 80%; PFS: 19.1 months.	[336]
		NY-ESO-1-LAGE-1 TCR-engineered T cells	II (NCT02992743)	NY-ESO-1	NY-ESO-1 positive advanced myxoid/​ round cell liposarcoma	CTX + FAMP + letetresgene autoleucel (GSK3377794).	ORR:30%.	[337]
		NY-ESO-1-LAGE-1 TCR-engineered T cells	I/II (NCT01567891)	NY-ESO-1	OC	NYESO-1^c259^ T cells	Completed.	Not found.
		NY-ESO-1 TCR-engineered T cells	II (NCT05549921)	NY-ESO-1	STS	NY-ESO-1-specific TCR-engineered T cells.	Recruiting.	Not found.
		MART-1 F5 TCR-engineered T cells	II (NCT00509288)	MART-1	Melanoma	CTX + FAMP + anti-MART-1 F5 TCR.	PR: 28.6%; PD: 71.4%.	[338]
		WT1 TCR-engineered T cells	I/II (NCT02550535)	WT1	MDS and AML	WT1 TCR transduced T cells.	Completed.	Not found.
		afamitresgene autoleucel	II (NCT04044768)	MAGE-A4	Advanced SS or myxoid/​round cell liposarcoma	afamitresgene autoleucel (previously ADP-A2M4).	ORR: 37%.	[339]
		Anti-MAGE-A3-DP4 TCR-engineered T cells	I/II (NCT02111850)	MAGE-A3	HLA-DP0401 positive metastatic cancer	CTX + FAMP + Aldesleukin + anti-MAGE-A3-DP4 TCR.	Completed.	Not found.
		MAGE-C2/HLA-A2 TCR-engineered T cells (MC2 TCR T cells)	I/II (NCT04729543)	MAGE-C2	Melanoma and head and neck cancer	MC2 TCR T cells.	Recruiting.	Not found.
		MAGE-A1 TCR-engineered T cells	I/II (NCT05430555)	MAGE-A1	Advanced solid tumors	Autologous CD8^+^ T-cells, transduced with MAGE-A1 directed TCR.	SD: 68.8%.	[340]
		E6 TCR-engineered T cells	I/I (NCT02280811)	HPV-16 E6	HPV associated cancers	CTX + FAMP + Aldesleukin + E6 TCR.	PR: 16.7%.	Not found.
		HBV TCR-engineered T cells	I (NCT03899415)	HBV	HCC	HBV TCR redirected T cells.	mOS: 33.1 months.	[341]
		P53 TCR-engineered T cells	II (NCT00393029)	P53	P53 overexpressed metastatic cancer	CTX + FAMP + Aldesleukin + Filgrastim + P53 TCR.	Completed.	Not found.
		Gavocabtagene autoleucel (TC-210) T Cells	I/II (NCT03907852)	Mesothelin	Advanced mesothelin-expressing cancer	FAMP + CTX + Nivolumab + Ipilimumab + gavo-cel.	ORR: 20%; CR:77%.	[342]
	TILs	TIL	III (NCT02278887)	-	Metastatic melanoma	FAMP + CTX + Ipilimumab + IL-2.	OR: 49% vs. 21%; mPFS: 7.2 months vs. 3.1 months; mOS: 25.8 months vs. 18.9 months (TIL group vs. Ipilimumab group).	[349]
		Lifileucel (LN-144)	II (NCT02360579)	-	Metastatic melanoma	Lifileucel.	ORR: 36%.	[350]
		LN-145	II (NCT04111510)	-	Metastatic TNBC	TIL LN-145.	Completed.	Not found.
		Young TIL	II (NCT01174121)	-	Metastatic cancer	FAMP + CTX + Pembrolizumab (Keytruda) + Aldesleukin + Young TIL.	CR: 2.4%; PR: 4.8%.	[351]
		Young TIL	II (NCT02133196)	-	Metastatic NSCLC	FAMP + CTX + Aldesleukin + Young TIL.	Recruiting.	[352]
		MDA-TIL	II (NCT03610490)	-	Multiple advanced solid tumors	FAMP + CTX + IL-2 + MDA-TIL.	mPFS: 2.53 months; mOS: 18.86 months.	Not found.

TIL: tumor-infiltrating lymphocyte, SCLC: small cell lung cancer, NSCLC: non-small cell lung cancer, MS: myelodysplastic syndromes, SCC: squamous cell carcinoma, SCCL: squamous cell carcinoma of the lung, ESCC: esophageal squamous-cell carcinoma, PC: pancreatic cancer, CC: colorectal cancer, EC: endometrial cancer, OC: ovarian cancer, GC: gastric cancer, ICC: intrahepatic cholangiocarcinoma, GBM: glioblastoma, mCRPC: metastatic castration-resistant prostate cancer, ccRCC: clear cell renal cell carcinoma, MDS: myelodysplastic syndrome, AML: acute myeloid leukemia, TNBC: triple-negative breast cancer, SS: synovial sarcoma, STS: soft tissue sarcoma, ORR: overall response rate, CR: complete responses, sCR: stringent complete response, PR: partial response, VGPR: very good partial response, SD: stable disease, PD: progressive disease, mPFS: median progression-free, mOS: median overall survival, MRD: minimal residual disease, TAA: tumor-associated antigens, Tec-Dara: Teclistamab in combination With daratumumab subcutaneously (SC), Tal-DP: Talquetamab SC in Combination With Daratumumab SC and Pomalidomide, Tal-D: Talquetamab SC in Combination With Daratumumab SC, DPd: Daratumumab SC, pomalidomide, and dexamethasone, DVd: Daratumumab SC, bortezomib, and dexamethasone, EPd: Elotuzumab, Pomalidomide, and Dexamethasone, CTX: cyclophosphamide, FAMP: fludarabine. data sourced from the *clinicaltrials.gov* site and the *chictr.org.cn* site

Despite the remarkable success of ICIs in cancers such as melanoma, non-small cell lung cancer, renal cell carcinoma, and Hodgkin lymphoma, responses remain heterogeneous, with a significant proportion of patients experiencing primary or acquired resistance [265–267]. Resistance mechanisms involve tumor-intrinsic factors, such as defective antigen presentation and interferon signaling, as well as immune-suppressive elements within the TME, including regulatory T cells, myeloid-derived suppressor cells, and alternative immune checkpoints [268]. To overcome these challenges, novel combinatorial strategies are under investigation, including ICIs paired with CAR T-cell therapy, cancer vaccines, and immune-modulating agents such as indoleamine 2,3-dioxygenase (IDO) inhibitors, STING agonists, and epigenetic modulators [269, 270]. Additionally, next-generation checkpoint inhibitors targeting emerging pathways, such as TIGIT and LAG-3, offer promising avenues for circumventing immune escape [271, 272].

Unlike inhibitory checkpoints, costimulatory molecules play a crucial role in T cell activation and function, making them promising therapeutic targets [273, 274]. mAbs targeting costimulatory receptors—including glucocorticoid-induced TNFR-related protein (GITR), 4-1BB, inducible T cell costimulator (ICOS), CD27, CD28, and OX40—are being evaluated in clinical trials [275, 276]. Despite their promise, agonist antibodies have yet to demonstrate significant clinical benefit, as most remain in early-phase trials. One exception was the ICOS-stimulatory monoclonal antibody feladilimab, which advanced to a phase III trial (NCT04428333) but was terminated due to a lack of clinical efficacy. As our understanding of tumor immunobiology deepens, combining immune checkpoint inhibitors with other immunotherapeutic strategies and targeting costimulatory pathways may further enhance antitumor immunity, ultimately leading to improved clinical outcomes for cancer patients.

#### T cell engagers (TCEs)

TCEs represent a transformative approach in cancer immunotherapy, leveraging T cells to eliminate malignant cells with high specificity [277–280]. These molecules bind both a tumor-associated antigen (TAA) and the CD3ε subunit of the TCR complex, enabling MHC-independent T-cell activation and cytotoxicity [281]. Unlike conventional monoclonal antibodies, TCEs do not require costimulatory signals, making them particularly effective in hematologic malignancies.

The clinical success of TCEs has been exemplified by blinatumomab (CD3×CD19), approved for rr-ALL. Additional FDA-approved TCEs include mosunetuzumab and epcoritamab-bysp (CD3×CD20) for follicular lymphoma, teclistamab and (CD3×BCMA) for multiple myeloma, and tarlatamab-dlle (CD3xDLL3) for extensive stage SCLC [282, 283] (Table 2). Beyond these, novel TCEs targeting CD38, CD123, and CLEC12A are in development for hematologic malignancies, while those against PSMA, EGFR, and HER2 are being explored for solid tumors [284–287] (Table 3). However, translating TCE efficacy to solid tumors remains challenging due to treatment-related toxicities and the immunosuppressive tumor microenvironment [288, 289].

The rapid evolution of bsAb engineering has led to the development of trispecific antibodies (TsAbs) and next-generation TCEs incorporating costimulatory domains (e.g., CD3×CD28, CD3 × 4-1BB) to enhance T-cell activation and persistence [290]. Dual-targeting checkpoint inhibitors, such as cadonilimab (PD-1×CTLA-4), have shown promise, while other bsAbs co-targeting immune checkpoints and TAAs (e.g., CD40×EpCAM) aim to improve tumor selectivity and reduce systemic toxicity [291–293]. Additionally, novel conditional TCEs with tumor-restricted activation and TCR-mimetic approaches, exemplified by tebentafusp-tebn (CD3xgp100) for uveal melanoma, represent key strategies to mitigate adverse effects and enhance efficacy [294–296].

Despite their promise, TCEs pose challenges such as CRS, ICANS, and on-target/off-tumor toxicity [289]. Strategies to optimize safety include step-up dosing regimens, affinity modulation, and tumor-specific activation mechanisms. Future directions involve combinatorial approaches with ICIs, CAR-T cells, and tumor microenvironment-modulating agents to overcome resistance and broaden the applicability of TCEs. Continued innovation in TCE design and clinical integration is expected to refine their therapeutic potential, advancing the landscape of cancer immunotherapy.

#### Adoptive T cell therapies

ACT has gained significant attention as an effective strategy for cancer immunotherapy. The approach involves the direct transfer of autologous or allogeneic T cells into patients, aiming to enhance the body’s immune response against tumors [297, 298]. ACT is generally classified into three main types based on the source of T cells and their mechanism of antigen recognition: CAR T cells, TCR-engineered T cells, and tumor-infiltrating lymphocyte (TIL) therapy. Each modality has its unique strengths, and ongoing advancements are refining these therapies to overcome existing challenges.

CAR T cell therapy is one of the most successful and widely used forms of ACTs, particularly for hematologic cancers [299–301]. CARs are genetically engineered proteins that enable T cells to recognize and target tumor-specific antigens. A typical CAR structure includes an extracellular antigen-binding domain (often a single-chain variable fragment, or scFv), a hinge region, a transmembrane domain, costimulatory domains (such as CD28 and 4-1BB), and an activation domain (commonly CD3ζ). These components work in concert to enhance T cell activation, proliferation, and effector functions [302, 303]. Over the past two decades, CAR T cell therapies have demonstrated remarkable success, particularly in hematologic malignancies like B cell lymphoma, leukemia and MM, with FDA-approved CAR T products targeting antigens such as CD19 and BCMA [304–313] (Tables 2 and 3). However, despite these successes, CAR T cells face significant limitations in solid tumors. Challenges include antigen heterogeneity, low tumor infiltration, and the immunosuppressive TME [34, 35, 314]. In response, next-generation CAR T cells are being developed to address these obstacles. Innovations include dual CAR constructs designed to target multiple antigens (e.g., CD19/CD20, CD19/CD22, CD19/BCMA, CD38/BCMA, CS1/BCMA, HER2/PD-L1) and logical gating systems that allow for more precise regulation of T cell activation [315–320]. Strategies are also focused on improving CAR T cell persistence and overcoming TME-induced inhibition, with promising results in preclinical and early-phase clinical trials [321]. An emerging strategy to enhance CAR T cell therapy is the combination of radiotherapy with CAR T treatment, which has shown unique synergistic effects in both hematologic malignancies and solid tumors [322–325]. Radiation therapy works by directly killing tumor cells through localized irradiation, thus reducing the number of target cells required for CAR T cell therapy and improving overall efficacy. Furthermore, radiation therapy modifies the TME by promoting the release of TAAs, upregulating MHC molecules, and triggering the activation of various immune cells [326–328]. These changes collectively enhance CAR T cell infiltration and activity, further improving therapeutic outcomes.

In contrast to CAR T cells, TCR-engineered T cells recognize intracellular tumor antigens presented on MHC molecules [329, 330]. This provides a significant advantage by expanding the range of tumor antigens that can be targeted, as TCR-T cells are capable of identifying peptides from intracellular proteins, which CAR T cells cannot. TCR T cells are also highly sensitive, requiring low antigen density for activation [331, 332]. However, TCR-T cells are limited by MHC restriction, which can restrict their applicability across diverse patient populations. Additionally, identifying the optimal tumor-specific TCRs and managing treatment-related toxicities, such as off-target effects, remain significant challenges [333, 334]. Early-phase clinical trials are ongoing to evaluate TCR-T cell therapies, with promising data on the use of TCRs targeting tumor-associated antigens like NY-ESO-1, MAGE-A, MART-1, Mesothelin and mutated neoantigens such as KRAS [335–342].

TIL therapy involves the adoptive transfer of tumor-specific T cells isolated directly from the tumor microenvironment. These T cells are typically expanded ex vivo to enhance their numbers before being reintroduced into the patient. TILs naturally exhibit a more effector-memory phenotype and are primed for migration to the tumor site, making them highly reactive against tumor cells [343, 344]. While TIL therapy has shown efficacy in solid tumors, particularly melanoma, challenges such as the limited number of TILs that can be extracted and expanded from tumors, as well as the need for patient-specific preparation, hinder widespread use [345–348]. Recent innovations, including the use of CD8^+^ enriched TILs and artificial APCs for expansion, have improved the scalability and efficacy of TIL therapies [349–352].

Despite the potential of adoptive T cell therapies, several challenges persist. One major concern is toxicity, particularly CRS and neurotoxicity, which are more prominent in CAR T cell therapies but can also affect TCR-T and TIL therapies. Mitigation strategies, such as IL-6 blockade and controlled dosing, are helping manage these toxicities [246, 353]. Additionally, antigen escape—where tumor cells lose or alter the targeted antigen—remains a major issue, particularly in CAR T cell therapies targeting surface antigens like CD19 [246, 354]. To address this, dual-targeting approaches and innovative Boolean logic CAR designs are under investigation to enhance specificity and reduce off-target toxicity [355]. Moreover, the immunosuppressive TME presents a significant barrier to the effectiveness of adoptive T cell therapies, especially in solid tumors. Various strategies, including the engineering of T cells resistant to TME-mediated suppression, are being explored [242]. Another major challenge lies in improving the persistence and expansion of engineered T cells within the body, which is critical for long-term anti-tumor immunity [356, 357].

Looking ahead, combination strategies involving adoptive T cell therapies with immune checkpoint inhibitors, targeted therapies, or even other forms of immunotherapy (e.g., monoclonal antibodies) may provide synergistic effects [321, 358, 359]. The development of allogeneic or “off-the-shelf” CAR T cells, which can be manufactured in advance and used for multiple patients, is another promising direction [244]. Additionally, advances in gene-editing technologies like CRISPR may allow for more precise modifications, further improving the efficacy and safety of these therapies [360]. Overall, adoptive T cell therapies, including CAR T cells, TCR T cells, and TILs, have revolutionized cancer immunotherapy. Although these therapies have shown remarkable promise, especially in hematological malignancies, their application to solid tumors and broader patient populations remains a work in progress. Ongoing innovations in T cell engineering, combination treatments, and better management of tumor resistance mechanisms are essential to overcoming the current limitations and expanding the clinical success of these therapies across a wide range of cancers.

#### Vaccines and T cell stimulation strategies

Cancer vaccines, particularly those targeting tumor-specific neoantigens, are an emerging form of therapeutic immunotherapy [361, 362]. Neoantigens are unique to individual tumors and are derived from genetic mutations, making them ideal targets for personalized cancer vaccines [363]. These vaccines aim to stimulate the immune system, especially T cells, to recognize and attack tumor cells [211, 364, 365]. While early clinical trials have shown promise, challenges such as the identification of optimal neoantigens and overcoming the immunosuppressive TME persist [366]. Combining neoantigen vaccines with immune checkpoint inhibitors may improve efficacy by enhancing T cell activation.

Adjuvants are crucial for enhancing the immune response to cancer vaccines by stimulating innate immunity and improving T cell activation [367]. Common adjuvants include Toll-like receptor (TLR) agonists, such as monophosphoryl lipid A (MPLA), and other immune modulators like CpG oligodeoxynucleotides and poly I: C. These adjuvants help activate dendritic cells, leading to better priming of T cells [368–371]. They are particularly useful in overcoming the suppressive TME in solid tumors, promoting T cell infiltration and function. Newer adjuvants, such as STING agonists, are also under investigation for their ability to enhance anti-tumor immunity [369, 372].

Combining cancer vaccines with immune checkpoint inhibitors, such as anti-PD-1 and anti-CTLA-4, has shown potential in boosting the efficacy of vaccines by blocking inhibitory signals and promoting T cell responses [373]. Additionally, combining vaccines with adoptive T cell therapies, such as CAR T or TCR-engineered T cells, may create a synergistic effect, enhancing both T cell priming and tumor-specific targeting [366, 374]. These combination approaches are aimed at improving the durability and specificity of anti-tumor immunity. In summary, neoantigen-based cancer vaccines, combined with adjuvants and other immunotherapies, offer a promising approach to enhancing T cell-mediated anti-tumor responses. While challenges remain, ongoing clinical trials are crucial for refining these strategies and improving their clinical effectiveness [375, 376].

#### Emerging technologies in T cell engineering

Recent advancements in T cell engineering are driving the evolution of immunotherapy, particularly for hematologic malignancies [377–380]. Innovations in gene editing, synthetic biology, and biomaterials are enhancing the precision, safety, and efficacy of these therapies [381–385]. CRISPR-Cas9, base editors, and prime editors allow for precise genetic modifications, such as optimizing TCRs and disrupting immune checkpoints to improve persistence and reduce exhaustion [386–389]. Multiplex gene editing is also enabling “off-the-shelf” allogeneic T cell therapies by eliminating endogenous TCRs and MHC molecules, reducing the risk of graft-versus-host disease (GVHD) [390–392].

Synthetic biology has introduced sophisticated control mechanisms to engineered T cells. Synthetic Notch (SynNotch) receptors and logic-gated CARs enhance targeting specificity, reducing toxicity and minimizing antigen escape [382, 393, 394]. Additionally, inducible gene circuits help regulate T cell activation, mitigating adverse effects such as CRS and neurotoxicity [395, 396]. These innovations improve both the safety and therapeutic durability of engineered T cells.

Advancements in biomaterials and cell manufacturing are further optimizing T cell expansion, persistence, and tumor infiltration [397, 398]. Nanoparticle-based delivery systems enhance the stability and bioavailability of therapeutic agents, improving in vivo efficacy [399, 400]. As these emerging technologies continue to evolve, addressing safety, durability, and regulatory challenges will be essential for the widespread clinical translation of next-generation engineered T cell therapies.

### Current challenges and future directions

T cells play a central role in anti-tumor immunity, but their therapeutic efficacy remains constrained by multiple challenges. One major obstacle is tumor immune evasion, which arises from both intrinsic and extrinsic mechanisms [6, 186, 314]. Tumors can escape immune detection by losing antigen expression, downregulating MHC molecules, or upregulating inhibitory ligands such as PD-L1. In parallel, the TME—rich in immunosuppressive components like Tregs, MDSCs, and inhibitory cytokines such as TGF-β and IL-10—actively impairs T cell activation, infiltration, and persistence. Although combination therapies and TME-modulating agents are under investigation, identifying context-specific drivers of immune exclusion and tailoring strategies accordingly remain significant knowledge gaps.

A second critical challenge is T cell exhaustion, a progressive state of dysfunction driven by chronic antigen exposure and sustained inhibitory signaling [11, 314, 401]. Exhausted T cells exhibit impaired proliferative capacity, reduced cytokine production, and upregulated inhibitory receptors such as PD-1, TIM-3, and LAG-3. Efforts to reverse exhaustion have focused on immune checkpoint blockade, metabolic reprogramming, and epigenetic modulation. However, durable reinvigoration of exhausted T cells remains a major hurdle, as transient functional restoration often leads to adaptation and therapy resistance.

Looking ahead, next-generation T cell therapies are being designed to improve persistence, trafficking, and cytotoxic function [402, 403]. Advances in gene editing technologies, particularly CRISPR/Cas9, allow for precise engineering of T cells—such as knocking out inhibitory genes or inserting synthetic signaling circuits—to boost functionality. However, the long-term safety and stability of such edits in patients remain to be rigorously evaluated. Similarly, novel CAR constructs with enhanced co-stimulatory domains or inducible cytokine release are under development, but optimal configurations for different tumor contexts have yet to be standardized. Another emerging strategy is TCR engineering, which enables targeting of intracellular tumor antigens presented via MHC, expanding therapeutic reach. Yet, the limited availability of shared neoantigens and the risk of off-target toxicity continue to pose major hurdles.

An exciting but still nascent area is the integration of systems biology and artificial intelligence to guide T cell therapy design [404–406]. Technologies like high-dimensional single-cell sequencing, proteogenomics, and machine learning algorithms are uncovering complex T cell differentiation states, exhaustion pathways, and resistance mechanisms. While these approaches hold great potential, translating such insights into actionable therapeutic interventions is still in its early stages and requires closer integration with clinical data and prospective validation.

In conclusion, although T cell-based therapies have transformed the landscape of cancer immunotherapy, key challenges—including immune evasion, exhaustion, and therapy resistance—still limit their full clinical impact. There remains a pressing need to deepen our understanding of the tumor–T cell interface, identify reliable biomarkers of durable response, and develop targeted interventions that can overcome the barriers outlined above. Continued innovation, supported by rigorous translational research and cross-disciplinary collaboration, will be essential for realizing the next generation of effective and personalized T cell therapies.

## Data Availability

No datasets were generated or analysed during the current study.

## Abbreviations

Treg: regulatory T cell
ACT: adoptive cellular immunotherapy
ICIs: immune checkpoint inhibitors
CAR: chimeric antigen receptor
CRS: cytokine release syndrome
ICANS: immune effector cell-associated neurotoxicity syndrome
GVHD: graft-versus-host disease
DN: double-negative
DP: double-positive
SP: Single-positive
TCR: T cell receptor
MHC: major histocompatibility complex
TME: tumor microenvironment
IL-7: interleukin-7
APCs: antigen-presenting cells
Th cells: CD4+ helper T cells
Tc cells: CD8+ cytotoxic T cells
Tfh cells: T follicular helper cells
Teff cells: Treg-to-effector T cells
ETPs: early thymic progenitors
CTLs: CD8+cytotoxic T lymphocytes
iNKT: invariant Natural killer T cells
MAIT: Mucosal-associated invariant T cells
BTN: butyrophilin
Fas-L: Fas ligand
DCs: dendritic cells
MR1: MHC-related protein 1
MDSCs: myeloid-derived suppressor cells
TAMs: tumor-associated macrophages
CAFs: cancer-associated fibroblasts
LAT cells: linker for activation of T cells
SLP-76: SH2 domain-containing leukocyte protein of 76 kDa
CSK: C-terminal Src kinase
DGKs: diacylglycerol kinases
AP1: activator protein 1
DAG: diacylglycerol
InsP3: inositol-1,4,5-trisphosphate
NFAT: nuclear factor of activated T cells
PKC: protein kinase C
PtdIns(4,5)P2: phosphatidylinositol-4,5-bisphosphate
RASGRP1: RAS guanyl-releasing protein 1
SKAP55: SRC kinase-associated phosphoprotein of 55 kDa
TCEs: T cell engagers
FDA: Food and Drug Administration
NMPA: National Medical Products Administration of China
HCC: hepatocellular carcinoma
mAbs: monoclonal antibodies
bsAbs: bispecific antibodies
TsAbs: trispecific antibodies
CSCC: cutaneous squamous cell carcinoma
r/r ALL: relapsed/refractory B cell precursor acute lymphoblastic leukemia
r/r FL: relapsed/refractory follicular lymphoma
r/r MM: relapsed/refractory multiple myeloma
SCLC: small cell lung cancer
NSCLC: non-small cell lung cancer
SSC: synovial sarcoma
NHL: non-Hodgkin’s lymphoma
DLBCL: diffuse large B-cell lymphoma
MCL: mantle cell lymphoma
MS: myelodysplastic syndromes
SCC: squamous cell carcinoma
SCCL: squamous cell carcinoma of the lung
ESCC: esophageal squamous-cell carcinoma
PC: pancreatic cancer
CC: colorectal cancer
EC: endometrial cancer
OC: ovarian cancer
GC: gastric cancer
ICC: intrahepatic cholangiocarcinoma
GBM: glioblastoma
mCRPC: metastatic castration-resistant prostate cancer
ccRCC: clear cell renal cell carcinoma
MDS: myelodysplastic syndrome
AML: acute myeloid leukemia
TNBC: triple-negative breast cancer
SS: synovial sarcoma
STS: soft tissue sarcoma
ORR: overall response rate
CR: complete responses
sCR: stringent complete response
PR: partial response
VGPR: very good partial response
SD: stable disease
PD: progressive disease
mPFS: median progression-free
mOS: median overall survival
MRD: minimal residual disease
Tec-Dara: Teclistamab in combination With daratumumab subcutaneously (SC)
Tal-DP: Talquetamab SC in Combination With Daratumumab SC and Pomalidomide
Tal-D: Talquetamab SC in Combination With Daratumumab SC
DPd: Daratumumab SC, pomalidomide, and dexamethasone
DVd: Daratumumab SC, bortezomib, and dexamethasone
EPd: Elotuzumab, Pomalidomide, and Dexamethasone
CTX: cyclophosphamide
FAMP: fludarabine
CTLA-4: cytotoxic T-lymphocyte-associated protein 4
PD-1: programmed death-1
PD-L1: programmed death-ligand 1
LAG-3: lymphocyte-activation gene 3
TIGIT: T cell immunoreceptor with Ig and ITIM domains
TIM-3: T cell immunoglobulin and mucin-domain containing-3
BTLA: B and T lymphocyte attenuator
VISTA: V-domain Ig suppressor of T cell activation
VEGF: vascular endothelial growth factor
IDO: indoleamine 2,3-dioxygenase
GITR: glucocorticoid-induced TNFR-related protein
ICOS: inducible T cell costimulator
TAA: tumor-associated antigen
TIL: tumor-infiltrating lymphocyte
TLR: Toll-like receptor
MPLA: monophosphoryl lipid A
SynNotch: Synthetic Notch
